# Dynamic and synchronous changes in metazoan body size during the Cambrian Explosion

**DOI:** 10.1038/s41598-020-63774-2

**Published:** 2020-04-22

**Authors:** Andrey Yu. Zhuravlev, Rachel Wood

**Affiliations:** 1 Department of Biological Evolution, Faculty of Biology, Lomonosov Moscow State University, Leninskie gory 1(12), Moscow, 119234 Russia; 2 School of GeoSciences, University of Edinburgh, James Hutton Road, Edinburgh, EH9 3FE UK

**Keywords:** Evolution, Solid Earth sciences

## Abstract

Many aspects of the drivers for, and evolutionary dynamics of, the Cambrian Explosion are poorly understood. Here we quantify high-resolution changes in species body size in major metazoan groups on the Siberian Platform during the early Cambrian (ca. 540–510 Million years ago (Ma)). Archaeocyath sponges, hyolith lophophorates, and helcionelloid mollusc species show dynamic and synchronous trends over million-year timescales, with peaks in body size during the latest Tommotian/early Atbadanian and late Atdabanian/early Botoman, and notably small body sizes in the middle Atdabanian and after the Sinsk anoxic extinction event, starting ca. 513 Ma. These intervals of body size changes are also mirrored in individual species and correlate positively with increased rates of origination and broadly with total species diversity. Calcitic brachiopods (rhynchonelliformeans), however, show a general increase in body size following the increase in species diversity through this interval: phosphatic brachiopods (linguliformeans) show a body size decrease that negatively correlates with diversity. Both brachiopod groups show a rapid recovery at the Sinsk Event. The synchronous changes in these metrics in archaeocyath, hyoliths and helcionelloids suggest the operation of external drivers through the early Cambrian, such as episodic changes in oxygenation or productivity. But the trends shown by brachiopods suggests a differing physiological response. Together, these dynamics created both the distinct evolutionary record of metazoan groups during the Cambrian Explosion and determined the nature of its termination.

## Introduction

The Cambrian Explosion, ca. 540–510 Ma records the dramatic diversification of metazoans and metazoan-dominated ecosystems, where the drivers may well be complex, with multiple feedbacks between dynamic environmental change and evolutionary response. Many typical early Cambrian groups such as archaeocyath sponges and hyoliths either went extinct or declined significantly during the Botoman-Toyonian extinction (ca. 513–508 Ma), starting with extensive shallow marine anoxia ca. 513 Ma, known as the ‘Sinsk Event’^1^. By contrast brachiopods and trilobites show modest changes in diversity across the Sinsk Event and continued to diversify thereafter^2^. The Sinsk Event hence effectively marks the end of the canonical Cambrian Explosion, and resets the trajectory for subsequent metazoan evolution.

Potential measures of the evolutionary dynamics across radiation events include temporal changes in biodiversity, varying origination and extinction rates, and changes in individual body size (soft tissue mass). Body size scales with many factors including individual metabolic rate, trophic status, temperature, and systematic affinity, and Cope’s rule (where lineages evolve larger body sizes over time), has been shown to be common over many taxa and long timescales^3^. This is not surprising as body size correlates with many measures of ecological success in benthic invertebrates, such as enhanced fecundity, competitive superiority, resistance to predation, and increased longevity, many of which can lead to preferential survival^4^. Indeed, a general increase in maximum metazoan body size is noted during the Ediacaran, Cambrian, and Ordovician periods (635–445 Mya)^5^. Counter wise, a body size reduction (the ‘Lilliput Effect’) is associated with episodes of mass extinction and ocean anoxia, and inferred oxygen stress [e.g. ^6^]. The general increase of maximum body size over time suggests a connection between complexity and size, but this relationship is relatively unexplored. In addition, we know little as to whether these changes are the result of passive or driven evolutionary processes^7^.

Surprisingly few high-resolution analyses of size evolution have been compiled from the Cambrian record even though size can be readily quantified and compared across fossil taxa. Here we quantify changes in species body size in skeletal metazoans from a single regional metacommunity – the Siberian Platform – through the early Cambrian, ca. 540 Ma to ~510 Ma. This records over half of known species through this interval, which are taxonomically and stratigraphically highly resolved and derived from a continuous succession of dominantly shallow marine carbonate sediments deposited at low latitude with minimal climatic fluctuations^8^.

## Materials and Methods

Maximum body size data were collated for species of cylindrical archaeocyath sponge cup diameter (n = 169), hyolith conch length (n = 185), helcionelloid mollusc aperture width (n = 79), brachiopod ventral valve width (n = 58), and siliceous sponge spicule length (n = 25) (see Supplementary methods and materials, data tables, and references [5–254]). We record the largest individual of each species in each time interval.

Cup diameter was chosen in archaeocyaths, as biovolume is not easy to quantify due their highly irregular cup shape and often cemented nature within a carbonate matrix. Hyoliths possess tubular conical conchs that commonly lack preservation of initial conch growth, so making biovolume estimation highly speculative. Helcionelloid molluscs and brachiopods possess almost equidimensional shells such that biovolume data would not show trends different from the linear dimensions. Hence in order to keep all data comparable we have used linear measures throughout.

Measurements are recorded to 10^th^ of a millimetre. Maximum sizes are taken from publications, except for archaeocyaths where cup diameters were recorded with a petrographic eyepiece micrometer from orientated thin sections, or using a metal graduated scale from outcrop. Molluscs were recovered via acid extraction from as steinkerns (internal conch moulds) of varying composition including glauconite and phosphate [e.g. ^9,10^] so minimising sampling and taphonomic bias.

Size data from early Cambrian trilobites was not compiled as the record is dominated by glabellar fragments that do not directly correlate with complete body size. Likewise, tommotiid, halkieriid, and other cataphract groups have only a few formal species for which the complete sclerotome has been reconstructed, or where a correlation between individual sclerite and full sclerotome size has been established^11^.

Three formations and their equivalents were sampled, namely the Pestrotsvet, Perekhod and Sinsk formations. The Pestrotsvet and Perekhod formations extend throughout the eastern region of the Siberian Platform and record a vast, non-restricted, fully marine carbonate platform lacking both evaporites and siliciclastics, except for some thin sandstone and siltstone units restricted to the basal Fortunian in the north-western region^12^. These successions are represented mainly by micritic/sparitic calcareous mudstone, and packstones to grainstones, with well-preserved bioclast, ooids and early marine cements. The Sinsk Formation by contrast shows an enrichment in redox-sensitive trace metals, a higher organic matter content, and a distinctive biomarker composition^1^.

We use the Siberian stratigraphic nomenclature with biostratigraphic zones combined with radiometric ages (Fig. S1). Officially, the Tommotian Stage is subdivided into three archaeocyath biozones^12^ but the middle biozone can be further subdivided into two subzones by small shelly fossils and archaeocyath assemblages^13^, and this is adopted here. These archaeocyath subzones can be traced around the entire Siberian Platform^14^.

We have not followed the current ICS timescale as this lacks definitive lower Cambrian subdivisions and all numerical ages, including the base of the Cambrian, are provisional. On the Siberian Platform, the first *Treptichnus pedum* appearance predates 541 Ma^15^, and recent dating of ash beds in the Nama Group, Namibia, indicates that the Ediacaran-Cambrian boundary could be younger than as 538 Ma^16^. Dating of Tommotian strata on the Siberian Platform that contains a Tommotian archaeocyath assemblages yields a 534.6 Ma age^17^ rather than the 529 Ma age based on the ICS timescale. Such issues make the strict application of the ICS timescale to any high resolution study highly problematic.

Origination and extinction rates were calculated in part by^18^ from the dataset of^1^ for the early Cambrian of the Siberian Platform. Extinction rate is the number of taxa that did not extend into an interval divided by total diversity, and origination rate is the number new taxa that appear in that interval divided by the total diversity. These macroevolutionary rates presented are regional rates such that speciation rate is a combination of true speciation and immigration, and extinction rates are a combination of true extinctions and extirpations.

### Temporal trends in body size of Cambrian metazoan groups in Siberia

**Figure 1:**
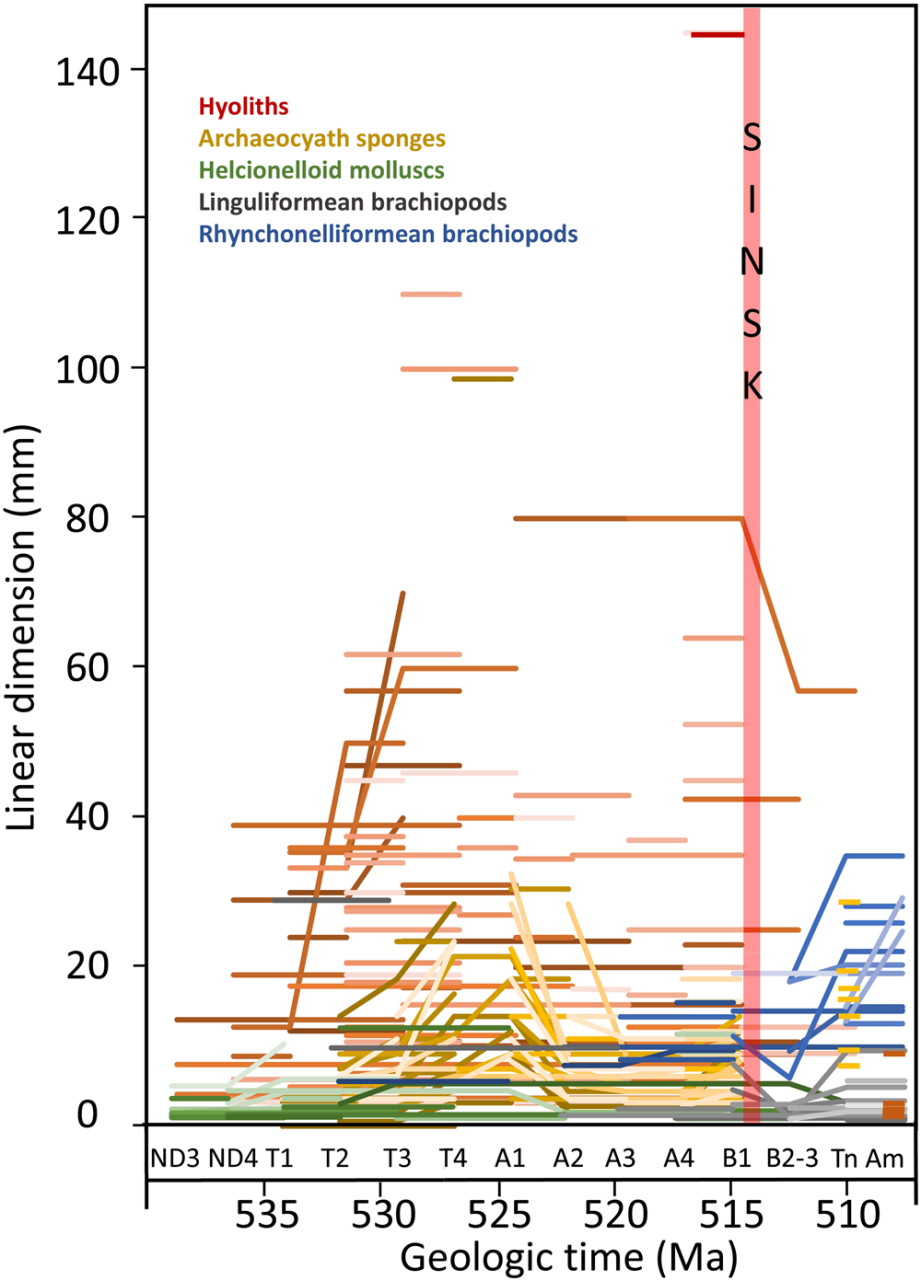
Temporal distribution of maximum species size (mm) for each time interval inhyoliths (conch length), cylindrical archaeocyath sponges (cup diameter), helcionelloid molluscs (aperture width), and linguliformean and rhyconelliformean brachiopods (ventral valve width) through the Early Cambrian on the Siberian Platform, with timing of the Sinsk Event^1^. ND = Nemakit-Daldynian; T = Tommotian; A = Atdabanian; B = Botoman; Tn = Toyonian; Am = Amgan.

**Figure 2:**
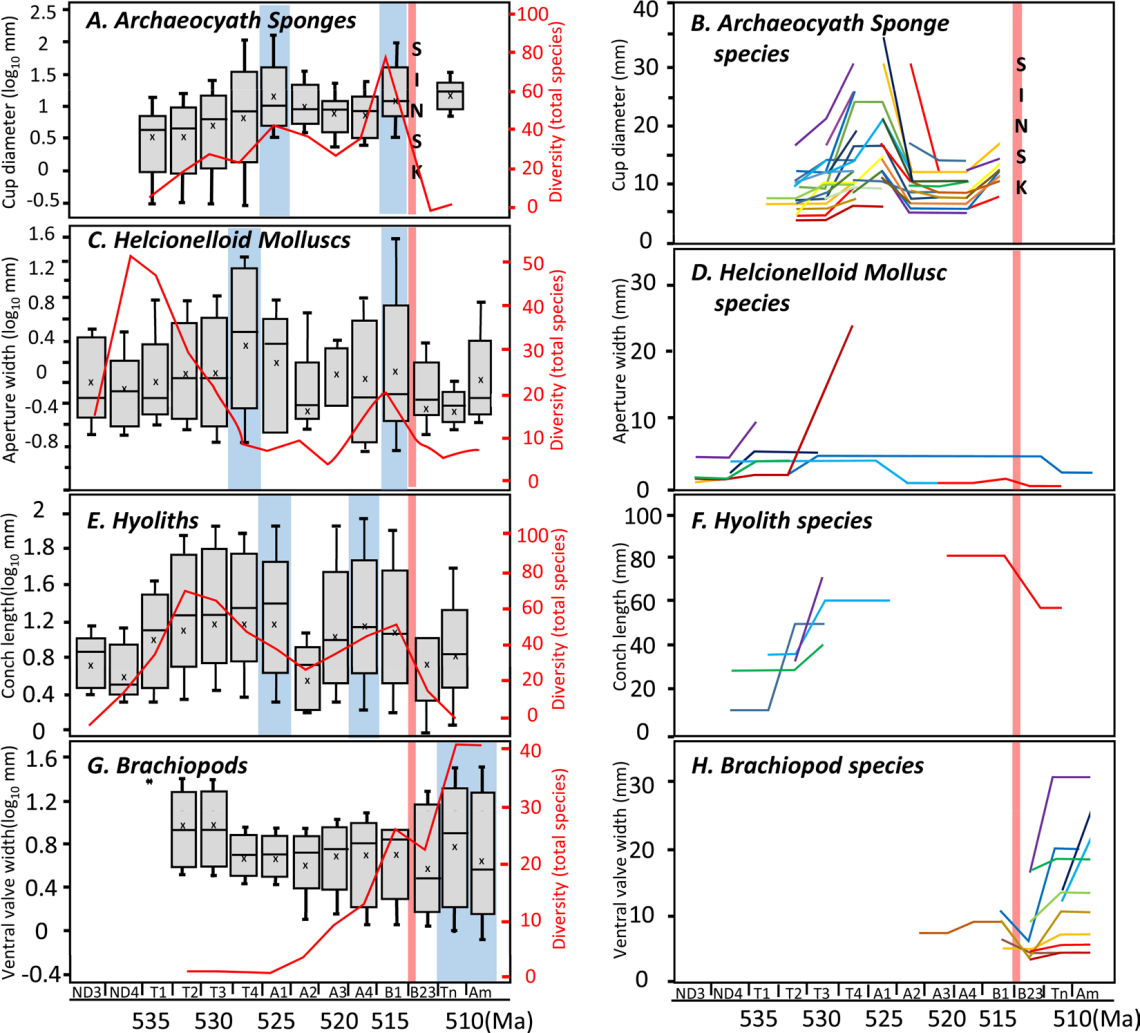
Box and whisker plots (log_10_ mm) showing changes in maximum species body size (**A,C,E,G**), and maximum size (mm) of individual species that change size through their range (**B,D,F,H**) (mm) through the Early Cambrian on the Siberian Platform, with total species diversity for each group (red line) and timing of the Sinsk Event^1^, in (**A**,**B**) Cylindrical archaeocyaths (cup diameter), (**C**,**D**), Helcionelloid molluscs (aperture width), (**E,F**) Hyoliths (conch length), **(G,H)** Brachiopods (ventral valve width). Data are shown with Maximum, 3^rd^ Quartile, Median (traced blue line), 1^st^ quartile and Minimum. ND = Nemakit-Daldynian; T = Tommotian; A = Atdabanian; B = Botoman; Tn = Toyonian; Am = Amgan. Maximum sizes reached for each group highlighted in blue.

Species show marked changes in maximum linear body size dimensions throughout the early Cambrian, but with no unidirectional trend through the interval (Fig. 1). Indeed all groups show marked oscillations of gradual body size increase and decrease (Figs. 1 and 2). Archaeocyath cup diameter shows peaks in the latest Tommotian to early Atdabanian, and again in the early Botoman (Fig. 2A). The Sinsk event resulted in the near extinction of the group, with only 8 species remaining by the Toyonian which are notably smaller than pre-Sinsk body sizes. Helcionelloid aperture width shows peaks in the latest Tommotian and again in the latest Atdabanian to early Botoman (Fig. 2C), and hyolith conch length also shows size peaks broadly in the same intervals (Fig. 2E). Both groups show reduced post-Sinsk Event body sizes. Maximum body size correlates positively with total species diversity in archaeocyaths and hyoliths, but with helicionelloids only after the Tommotian (Fig. 2A,C,E).

**Figure 3:**
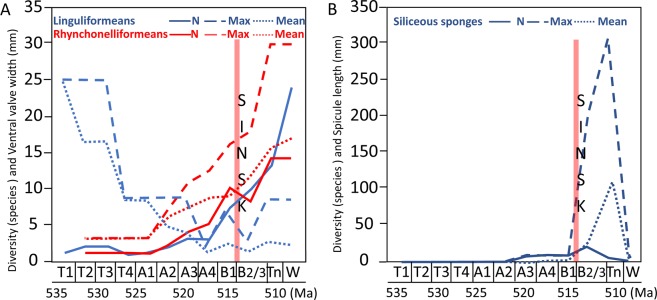
Changes in species diversity, and maximum, minimum and mean body size (mm) through the Early Cambrian on the Siberian Platform with timing of Sinsk Event^1^, in (**A**) Linguliformean (phosphatic) and rhynchonelliformean (calcitic) brachiopods (ventral valve width), and (**B**) Siliceous sponge spicules (length). T = Tommotian; A = Atdabanian; B = Botoman; Tn = Toyonian; W = Wuliuan.

Brachiopods show very different trends. As a group, size is high during the Tommotian, low in the Atdabanian but rising slightly until the early Botoman, then increasing after the Sinsk Event, with no correlation with total species diversity (Fig. 2G). Phosphatic linguliformeans and calcitic rhynchonelliformeans, however, show opposing metrics. Linguliformeans display no positive correlation of maximum size with diversity, but rhynchonelliformeans show a very strong correlation (Fig. 3A). Only linguliformeans show only a temporary drop in body size after the Sinsk Event.

Siliceous sponge spicules show a different pattern, with low diversity and small spicule length (<10 mm) throughout the Tommotian to Atdabanian interval, but a marked increase in diversity and spicule size after the Sinsk Event in the later Botoman and Toyonian (Fig. 3B).

### Temporal body size changes in individual species

Of those archaeocyath species (n = 83) that appear in more than one stratigraphic unit (i.e. non-singletons), 44 species (53%) show dramatically changing cup diameters though their ranges, in some cases more than doubling in size (Fig. 2B). All these individual species synchronously follow the same group trends in size, with a marked increase in size from the mid to late Tommotian, a decrease from the early to mid Atbadanian, and a further increase from the late Atdabanian to early Botoman. Although only 5 species show a continuous record across the late Tommotian to early Atdabanian interval, many taxa probably represent closely related or over-split species. Although the number of species that change size through their ranges is far smaller, both molluscs (n = 9) and hyoliths (n = 5) follow broadly similar trends (Fig. 2D,F). Brachiopod species that change size through their ranges (n = 12) show a size decline after the Sinsk but a rapid increase thereafter (Fig. 2H).

### Temporal trends in early Cambrian metazoan origination and extinction rates

**Figure 4:**
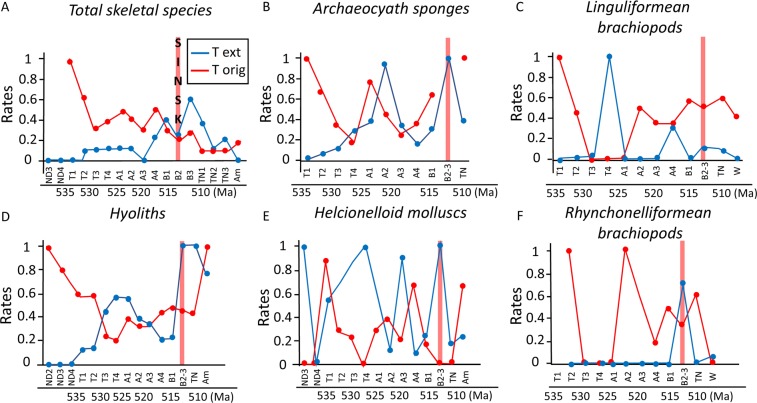
Changing species origination and extinction rates through the Early Cambrian on the Siberian Platform with timing of Sinsk Event, for (**A**) Total skeletal taxa; (**B**) Archaeocyath sponges; (**C**) Linguliformean (phosphatic) brachiopods; (**D**) Hyoliths; (**E**) Helcionelloid molluscs; (**F**) Rhynchonelliformean (calcitic) brachiopods. ND = Nemakit-Daldynian; T = Tommotian; A = Atdabanian; B = Botoman; Tn = Toyonian; Am = Amgan; W = Wulian. Data from^1^, in part calculated from^18^.

The dramatic fluctuations in diversity during the Cambrian Radiation result from high rates of both origination and extinction. Total skeletal species (n = 1188) show generally declining rates of origination through the early Cambrian, and elevated extinction rates during the Botoman, which was also a period of low origination rates, in part coinciding with the Sinsk Event (Fig. 4A).

Individual groups show differing dynamics. Hyoliths (n = 214), archaeocyaths (n = 205), and helcionelloids (n = 79) show high origination rates in the late Nemakit-Daldynian to early Tommotian, early Atdabanian, and over the mid-Atdabanian to early Botoman interval (Fig. 4B,D,E). Elevated extinction rates occur in the late Tommotian to mid-Atdabanian, and mid-Botoman. Brachiopod species (n = 93), however, present notably opposing origination dynamics, with both linguliformeans and rhynchonelliformeans showing peaks of origination in the early Tommotian, mid Atdabanian, and after the Sinsk Event (Fig. 4C,F). Only rhynchonelliformeans display a minor peak of extinction coincident with the Sinsk Event.

## Discussion

Sessile archaeocyath sponges, semi-sessile hyolith lophophorates, and motile helcionelloid molluscs show dynamic and congruent changes in body size despite their differing biology and ecology, and all were decimated by the Sinsk Event. All these groups show a prominent size increase during the latest Tommotian/earliest Atdabanian and latest Atbabanian/earliest Botoman, and a marked decrease during the mid-Atdabanian. Body size changes correlate with enhanced rates of origination, as well as individual species size trends and generally with total species diversity. All these groups show markedly smaller body sizes after the Sinsk Event, the latter interpreted here as a post-extinction ‘Lilliput Effect’. This effect is not solely the result of the preferential loss of larger species, as some species that survived also show body reduction (Fig. 2D,F).

Brachiopod trends are notably divergent. Brachiopods as a group show no decrease in diversity or a ‘Lilliput Effect’ during and after the Sinsk Event, but a few individual linguliformean and rhynchonelliformean species exhibit a transient size reduction (Fig. 2H). Linguliformeans display no positive correlation of maximum size with diversity, but rhynchonelliformeans show a very strong correlation. While hyolith and helcionelloid conchs are scarce in the Siberian Sinsk Formation, brachiopod shells are reasonably abundant^19^. In addition, the maximum and mean sizes of the first known brachiopods, which were linguliformeans, were larger than the majority of other early Cambrian species, including the first rhynchonelliformeans, which also appeared later. Here large size may be inherited from their ancestors, which may have been either tommotiids or large pedunculate hyolith-like forms^11^.

These dynamic size changes over Myr timescales counter the suggestion that the small shell dimensions of early Cambrian molluscs are a preservational or sampling artefact^20,21^. Indeed, the synchronous changes noted in archaeocyaths, hyoliths and helcionelloids suggest the operation of an external driver. Modern marine benthic invertebrate communities show a markedly decreased size structure in response to the reduced oxygen availability^22^, but correlation between oxygen levels and size is complicated by the covariation of oxygen with variables such as temperature, food availability, and the competing effects of other co-habiting species. Well-oxygenated waters are associated with larger body sizes, higher diversity, complex skeletal biomineralization, and increased motility and predation^23–26^. The Siberian Platform shows climatic stability throughout the Early Cambrian, but we suggest that pulses of oxygenation and productivity, and intervening intervals of anoxia, may have influenced the evolution of body size.

A general rise in oceanic oxygen has been invoked to explain the Cambrian Explosion, but the exact role of oxygen as a driver for early animal evolution remains unclear^27^. The nature of the physiological response of metazoans to changes in oceanic oxygen has not been established, with different groups and metabolisms potentially having differing oxygen demands and hence responses depending on size, mobility, nervous and circulation systems, and ecology^28^.

**Figure 5:**
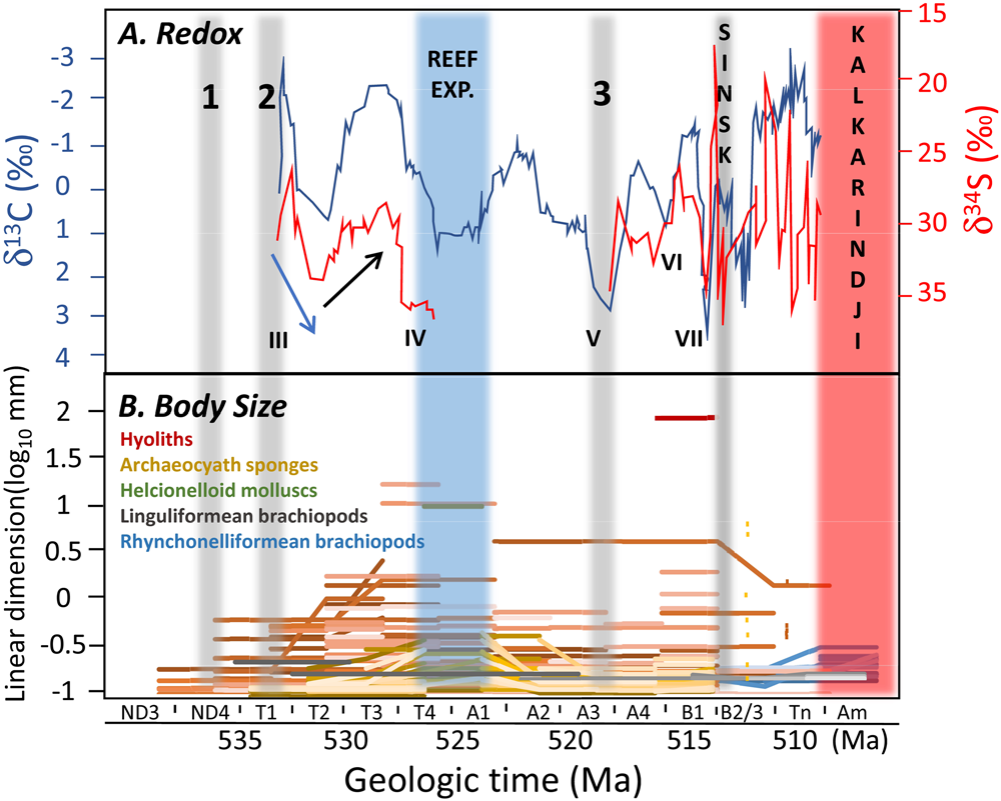
(**A**) Proposed changes in redox through the Lower Cambrian on the Siberian Platform, with timing of the Sinsk anoxic^1^ and Kalkarindji flood basalt events^42^. Other inferred global anoxic events 1, 2^31^, and 3;^30^ Carbon and sulphur isotope data and cycles III-VII on the Siberian Platform^35^. Blue arrow indicate inferred oxygenation of more anoxic oceans; black arrow de-oxygenation of more oxic oceans. Reef Exp. = Reef expansion from^36^. (**B**) Temporal distribution of maximum species body size (log_10_ mm) for each time interval in hyoliths (conch length), cylindrical archaeocyath sponges (cup diameter), helcionelloid molluscs (aperture width), and linguliformean and rhyconelliformean brachiopods. ND = Nemakit Daldynian; T = Tommotian; A = Atdabanian, B = Botoman; Tn = Toyonian; Am = Amgan.

Several studies have proposed that the early Cambrian was a time of dynamic oxygenation, but existing data are in notable contradiction (Fig. 5A). Some models suggest an overall trend of oxygenation caused by rising atmospheric pO_2_ of up to ~0.3 present atmospheric level (PAL) by the early Cambrian^29^. Uranium isotopes suggest episodes of globally expanded anoxic waters during the Ediacaran-Cambrian transition and late Terreneuvian based on data from South China^30^. Similar data from Siberia, Morocco, and South China combined with mass balance models^31^ suggests a further two preceding phases of expanded seafloor anoxia coincident with positive carbon isotope excursions during the early and late Terreneuvian, where it is suggested that atmospheric pO_2_ levels may have declined in concert with global new marine production and enhanced phosphorous burial in bioturbated sediments. Based on radiometric dating and the first appearance datum of *Watsonella crosbyi* on the Siberian Platform^32^, these early anoxic intervals correlate with the late Nemakit-Daldynian and early Tommotian, although significant difficulties remain in correlation between different regions. Both these anoxic phases predate the synchronous size increases noted here, and the first interval may coincide with the small size noted in hyoliths and helcionelloids in the latest Nemakit-Daldynian (Fig. 2C,E), and the second interval with small archaeocyath sizes in the earliest Tommotian (Fig. 2A). We also note that increased extinction rates peak during the middle Meichucunian and Canglangpuan in South China when euxinic and/or anoxic waters were widespread in marine basins^33,34^.

The early Cambrian record on the Siberian Platform shows transient (0.5–2 Myr) coupled carbonate δ^13^C and carbonate-associated sulphate δ^34^S cycles (III-VII)^35^ (Fig. 5A). In contrast to^31^, biogeochemical modelling of these cycles suggests that each positive carbon isotope excursions represents a pulse of oxygenation within highly productive anoxic oceans with relatively low sulphate concentration^35^. Additional positive feedbacks between ocean ventilation, phosphorus retention in sediments, and biological ventilation behaviour, may have also driven rapid bottom-water oxygenation leading to the re-establishment of anoxia so potentially creating the repetitive cycles. These extreme oxygen perturbations would have created temporarily expanded shallow habitable shelves, and an increased diversity of metazoans is inferred to have coincided with these intervals^35^. The Sinsk Event coincided with decoupling of the isotope records, suggesting a further shrinking of the marine sulphate reservoir, as well as expanded shallow marine anoxia.

Maximum sizes in archaeocyaths, hyoliths and helcionelloids correspond to the most positive δ^13^C and δ^34^S values where models suggest the highest rates of O_2_ production and productivity (Fig. 5B). The largest archaeocyaths have short ranges (<2 Myr) and appear only during the modelled height of the atmospheric oxygenation peak during cycle IV. In hyoliths and helcionelloids, the first peak in maximum size appears slightly earlier, but nonetheless is broadly coincident with cycles IV and V-VII. The late Tommotian/early Atdabanian is also an interval of widespread reef diversification on the Siberian Platform, when the diversity of community types dramatically increased as shown by elevated beta-diversity^36^. Elevated rates of origination occur in hyoliths and helcionelloids in cycle IV, and in helcionelloids only in cycle V.

Varying oxygen tolerance due to differences in hard-parts, mobility, and metabolic rates and differences in response to productivity may have determined both the differential trends of the groups interrogated here through the early Cambrian as well as their fate at the Sinsk Event. Archaeocyaths show highly dynamic size changes over Myr timescales, and it is possible that skeletal growth was promoted by atmospheric oxygen and/or productivity pulses. It is unlikely that archaeocyaths differed from other sponges in their food requirements and metabolic rates, but they nonetheless show a very different temporal size distribution to siliceous sponges as inferred by spicule dimensions (Figs. 2A and 3B). This may be due to the additional energy required to form a heavily calcified skeleton (hypercalcification). The formation of calcified skeletal tissue in all animals requires collagenous template biosynthesis and a sufficient energy supply for crystal nucleation^26^ but a reduction of surface-water carbonate supersaturation during phases of enhanced anoxia would also make skeletal formation in hypercalcifiers more difficult^37^. Hypercalcified reef-building sponges are noted to decline during the end-Devonian and Permo-Triassic mass extinctions, also in part ascribed to expanded anoxia and reduced surface-water aragonite supersaturation^38^. Calcareous helcionelloid molluscs follow other calcifiers in their size distribution, but a similar biomineral discrepancy is also observed in the temporal size distribution and origination/extinction rates of the two lophophorate groups - hyoliths with calcareous conchs, and linguliformean brachiopods with phosphatic shells.

Molluscs with the same external linear dimensions as brachiopods have two-three times greater oxygen consumptions^39,40^. This may explain the continued diversification of brachiopod species during the Sinsk Event. The Sinsk Event led to near extinction of many calcifiers, probably as a result of limited tolerance to low oxygen and hypercapnia. While all the calcifying groups display the ‘Lilliput Effect’ in the aftermath of the Sinsk Event, both phosphatic linguliformean brachiopods and siliceous sponges manifest the opposite ‘Brobdingnag Effect’ - the within‐species size increase of newly originated species^41^. The latter part of the Botoman-Toyonian extinction was coeval with emplacement of the Kalkarindji Large Igneous Province at 508–498 Ma^42^. This event, although potentially detrimental to the latest early Cambrian reef biota, post-dated the major animal size changes recorded here which occurred before 510 Ma.

In sum, these data show that neither Cope’s Rule nor any other ‘rules’ explaining body size changes apply consistently to the early Cambrian record of metazoans on the Siberian Platform. Species show highly dynamic and, in several groups, broadly synchronous changes in body size over Myr timescales throughout this interval. This suggests the operation of extrinsic, but episodic, driving evolutionary processes. We propose here that these may be fluctuating shallow marine oxygen levels and/or productivity pulses. But the differing responses of other groups suggests a possible role for physiology and the cost of calcification. In so doing, this created both the distinct record of early metazoans in this metacommunity during the Cambrian Explosion, and also the form of its demise.

## Supplementary information

Supplementary information.

Supplementary information 2.
