# Supplementary material for: Dynamic and synchronous changes in metazoan body size during the Cambrian Explosion
Source: Sci Rep. 2020 Apr 22;10:6784. doi: 10.1038/s41598-020-63774-2 (PMC7176670; doi:10.1038/s41598-020-63774-2)
Supplement: Supplementary file 1 — Supplementary information. [file 41598_2020_63774_MOESM1_ESM.docx]

**Dynamic and synchronous changes in metazoan body size during the Cambrian Explosion**

**Supplementary Information and Data**

**Andrey Yu. Zhuravlev^1^, Rachel A. Wood^2*^**

**^1^**Department of Biological Evolution, Faculty of Biology, Moscow State University named

after M.V. Lomonosov, Moscow GSP-1, 119991, Russia

**^2^**School of GeoSciences, University of Edinburgh, King's Buildings, James Hutton Road,

Edinburgh EH9 3FE, UK. [*Rachel.Wood@ed.ac.uk](mailto:*Rachel.Wood@ed.ac.uk)

**Materials and Methods, with References 1-4**

**Data Tables (.xlsx)
Fig. S1
References 5-254**

**Materials and Methods**

Most variation in body size is among species and genera rather than within them [1,2], and the sizes of illustrated specimens typically correlates with the mean and maximum of populations from bulk samples [3,4]. The sizes of specimens can therefore be used to evolutionary trends. For each species, we measured a major axis using the specimens illustrated in the literature (see Supplementary references [5-254]), and assigned each to the stratigraphic units in Supplementary Figure 1. For maximum completeness, we used the longest linear dimension of the largest specimen for each species in all calculations. In archaeocyaths we chose cup diameter as this is recorded more often in the literature than cup height, as it is more readily measured in the field and in thin section. For hyoliths we chose conch length as this is most commonly reported. For similar reasons we report aperture width in helcionelloid molluscs and ventral valve width in brachiopods.

**Possible taphonomic and sampling biases**. Archaeocyaths sponges are readily studied and quantified in outcrop or thin section, and are restricted to shallow marine carbonate facies.

Ediacaran to Cambrian skeletal lophotrochozoans (including hyoliths, helcionelloids and brachiopods) are represented by taxa of comparable millimetric sizes, forming part of the small shelly fauna as shells and disarticulated sclerites. These fossils are generally either replaced by phosphate or present in the form of inner and outer moulds. Only lingulate brachiopods and tommotiids are preserved as original shells, and only rhynchonelliform brachiopods retain their original low-Mg calcite mineralogy. In the lower Cambrian of the Siberian Platform, such fossils are restricted to argillaceous limestones (mostly wackestones and packstones), and some grainstones, all of which accumulated onshore above either normal wave or storm wave base. All fossils are extracted by the same method of dissolution in buffered acetic acid to isolate phosphatic and phosphatized shells, or moulds and steinkerns. Worker bias is unlikely given that the assemblages reflect multiple different studies and no single worker or study dominates.

We infer that taphonomic biases are minimized, and sampling biases present are shared by all studied skeletal fossils.

1. Rego, BL, Wang, SC, Altiner, D, Payne, JL. Within- and among-genus components of size evolution during mass extinction, recovery, and background intervals: a case study of Late Permian through Late Triassic foraminifera. *Paleobiology* **38**, 625–641 (2012)
2. Smith, FA et al. 2004 Similarity of mammalian body size across the taxonomic hierarchy and across space and time. *Am. Nat*. **163**, 672–691 (2004).
3. Krause, RA, Stempien, JA, Kowalewski, M, Miller, AI. Body size estimates from the literature: utility and potential for macroevolutionary studies. *Palaios* **22,** 60–73 (2007).
4. Kosnik MA, Jablonski D, Lockwood R, NovackGottshall PM. 2006 Quantifying molluscan bodysize in evolutionary and ecological analyses: maximizing the return on data-collection efforts. Palaios 21, 588–597 (2206).

Data tables (.xlsx) of species distribution through the early Cambrian with maximum size achieved for each time interval (mm), at start of each interval. n/a = no data available.

ND = Nemakit Daldynian; T = Tommotian; A = Atdabanian; B = Botoman; Tn = Toyonian; Am = Amgan; W = Wuliuan.


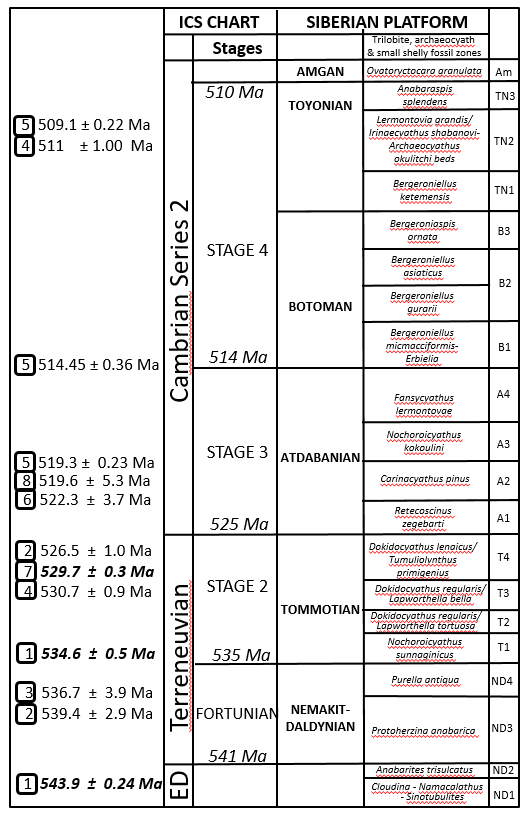


***Supplementary Fig. 1: Siberian timescale with biozones, ICS and Siberian stages, and correlated radiometric dates.* *Modified from [1].***

**Supplementary References for quantitative temporal distribution of skeletal taxa on the Siberian platform**

1. Aksarina, N.A. & Pel’man, Yu.L. (Cambrian Brachiopods and Bivalved Molluscs of Siberia). *Institut Geologii i Geofiziki Sibirskogo Otdeleniya Akademii Nauk SSSR, Trudy* **316**, 1–178 (in Russian) (1978).
2. Andreeva, O.N. (Cambrian artriculate brachiopods). *Paleontologicheskiy zhurnal* **1987 (4),** 31–40 (in Russian) (1987).
3. Andreeva, O.N. (On some Cambrian-Ordovician brachiopods of the Siberian Platform). *Ezhegodnik Vsesoyuznogo Paleontologicheskogo Obshchestva* **32**, 4–76 (in Russian) (1989).
4. Astashkin, V.A., Pegel’, T.V., Repina, L.N., Rozanov, A.Yu., Shabanov, Yu.Ya., Zhuravlev, A.Yu., Sukhov, S.S. & Sundukov, V.M. 991. The Cambrian System on the Siberian Platform. Correlation chart and explanatory notes. *International Union of Geological Sciences, Publication* **27,** 1–133 (1991).
5. Astashkin, V.A., Varlamov, A.I., Egorova, L.I. & Shabanov, Yu.Ya. (Stratigraphic position of trilobites of the “Sanashtykgol” assemblage in the stratotype section of the Lower Cambrian of the Lena River). *Sibirskiy Nauchno-Issledovatel’skiy Institut Geologii, Geofiziki i Mineral’nogo Syr’ya, Trudy* **287**, 5–15 (in Russian) (1981).
6. Barskova, M.I. (New species of Lower Cambrian gastropods from the Uchur-Maya region). *Paleontological Zhurnal* **1987 (2),** 124–127 (in Russian) (1987).
7. Barskova, M.I. (New molluscs from the Lower Cambrian strata of the Kolyma Uplift). *Paleontological Zhurnal* **1988 (1),** 101–105 (in Russian) (1988).
8. Bengtson, S. The Lower Cambrian fossil *Tommotia*. *Lethaia* **3**, 363–392 (1970).
9. Bengtson, S. Early Cambrian button-shaped phosphatic microfossils from the Siberian Platform. *Palaeontology* **20**, 751–762 (1977).
10. Bengtson, S., Matthews, S.C. & Missarzhevsky, V.V. The Cambrian netlike fossil *Microdictyon*. In A. Hoffman & M.H. Nitecki, eds. *Problematic Fossil Taxa*, pp. 97–115. New York, Oxford University Press; Oxford, Clarendon Press (1986).
11. Bengtson, S., Fedorov, A.B., Missarzhevsky, V.V., Rozanov, A.Yu. & Zhuravlev, A.Yu. *Tumulduria incomperta* and the case for Tommotian trilobites. *Lethaia* **20**, 361–370 (1987).
12. Bokova, A.R. (The oldest assemblage of Cambrian organisms of western Prianabar’e). In V.V. Khomentovsky, ed. (Stratigraphy of the Late Precambrian and Early Palaeozoic of Siberia. Vendian and Riphean), pp. 13-28. Novosibirsk, Institut *Geologii i Geofiziki Sibirskogo Otdeleniya Akademii Nauk SSSR* (in Russian) (1985).
13. Bokova, A.R. (New Lower Cambrian gastropods of the Siberian Platform). *Paleontological Zhurnal* **1990 (2),** 123–126 (in Russian) (1990).
14. Bushuev, E., Goryaeva, I. & Pereladov, V. New discoveries of the oldest trilobites *Profallotaspis* and *Nevadella* in the northeastern Siberian Platform, Russia. *Bulletin of Geosciences* **89**, 347–364 (2014).
15. Chernysheva, N.E. (Cambrian Strata of the Upper Priangar’e, Their Fauna and Position in the Generalised Section of the Cambrian of the Central Parts of the Siberian Platform (Yakutia)). *Irkutsk, Vostsibneftegeologiya.* 44 pp (in Russian). (1950).
16. Chernysheva, N.E. (Stratigraphy of the Cambrian of the Aldan Anteclise and the palaeontological grounds for distinguishing the Amgan Stage). *Vsesoyuzniy Nauchno-Issledovatel’skiy Geologicheskiy Institut, Trudy (Novaya Seriya)* **49**, 1–347 (in Russian) (1961).
17. Chernysheva, N.E. (Cambrian trilobites of the family Oryctocephalidae). *Nauchno-Issledovatel'skiy Institut Geologii Arktiki, Trudy* **127**, pp. 3–52 (in Russian) (1962).
18. Datsenko, V.A., Zhuravleva, I.T., Lazarenko, N.P., Popov, Yu.N. & Chernysheva, N.E. (Biostratigraphy and fauna of the Cambrian deposits of the northwestern Siberian Platform). *Nauchno-Issledovatel’skiy Institut Geologii Arktiki, Trudy* **155**, 1–213 (in Russian) (1968).
19. Debrenne, F., Lafuste, J. & Zhuravlev, A. Coralomorphes et spongiomorphes a l'aube du Cambrien. *Bulletin du Muséum national d‘Histoire naturelle, Paris,* 4e série **12**, 17–39 (1990).
20. Debrenne, F., Rozanov, A. & Zhuravlev, A. Regular Archaeocyaths. *Éditions du Centre National de la Recherche Scientifique, Cahiers de Paléontologie.* Paris. 218 pp (1990).
21. Debrenne, F, & Zhuravlev, A. Irregular Archaeocyaths. *Éditions du Centre National de la Recherche Scientifique, Cahiers de Paléontologie.* Paris. 212 pp (1992).
22. Debrenne, F., Zhuravlev, A.Yu., & Rozanov, A.Yu. (New genera of regular tabular and single-chambered archaeocyaths from the Lower Cambrian of Siberia). *Paleontologicheskiy Zhurnal* **1988 (4),** 97–99 (in Russian) (1988).
23. Demidenko, Yu. E. New Cambrian lobopods and chaetognaths of the Siberian Platform. *Paleontological Journal* **40,** 234–243 (2006).
24. Demidenko, Yu. E. Morphology, taxonomic position, and stratigraphic distribution of the Early Cambrian skeletal problematic *Mobergella radiolata* Bengtson, 1968. *Paleontological Journal* **50**, 435–449 (2016).
25. Demokidov, K.K. & Lazarenko, N.P. (New data on the stratigraphy of Cambrian strata of the western slope of the northern Kharaulakh), In N.A. Shvedov, ed. (*Collection of Papers on Palaeontology and Stratigraphy*, Issue **16**), pp. 11–22. Leningrad, NIIGA (in Russian) (1959).
26. Demokidov, K.K. & Lazarenko, N.P. (Upper Precambrian and Cambrian stratigraphy and the Lower Cambrian trilobites of the northern part of Middle Siberia and the Soviet Arctic islands). *Nauchno-Issledovatel'skiy Institut Geologii Arktiki, Trudy* **137**, 1–288 (in Russian) (1964).
27. Dzik, J. Evolution of ‘small shelly fossils’ assemblages. *Acta Palaeontologica Polonica* **39**, 247–313 (1994).
28. Egorova, L.I. (Some Lower and Middle Cambrian trilobites of the Siberian Platform). *Paleontologicheskiy Zhurnal* **1967 (1),** 68–78 (in Russian) (1967).
29. Egorova, L.I. (New Middle Cambrian trilobites of the north of the Siberian Platform). *Paleontologicheskiy Zhurnal* **1970 (4),** 72–76 (in Russian) (1970).
30. Egorova, L.I. (Cambrian trilobites of the east of the Siberian Platform and their facies restriction). *Sibirskiy Nauchno-Issledovatel’skiy Institut Geologii, Geofiziki i Mineral’nogo Syr’ya, Seriya Regional’naya Geologiya, Trudy* **110,** 39–46 (in Russian) (1970).
31. Egorova, L.I. (New Lower Cambrian trilobites of the southeastern Siberian Platform). *Paleontologicheskiy Zhurnal* **1983 (3),** 59–64 (in Russian) (1983).
32. Egorova, L.I. & Korobeynikova, T.V. (Trilobite assemblages of the type sections of the Obruchev and Elanka horizons from the Lower Cambrian of Siberia). *Sibirskiy Nauchno-Issledovatel’skiy Institut Geologii, Geofiziki i Mineral’nogo Syr’ya, Trudy* **192**, 35–45 (in Russian) (1974).
33. Egorova, L.I. & Savitskiy, V.E. (Cambrian stratigraphy and biofacies of the Siberian Platform. Western Anabar area). *Sibirskiy Nauchno-Issledovatel’skiy Institut Geologii, Geofiziki i Mineral’nogo Syr’ya, Trudy* **43**, 1–408 (in Russian) (1969).
34. Egorova, L.I. & Shabanov, Yu.Ya. (Cambrian trilobites on the north of the Anabar-Sinsk facies region of the Siberian Platform). In S.P. Bulynnikova & I.G. Klimova, eds. (*New Species of Ancient Plants and Invertebrates from the Phanerozoic of Siberia*), pp. 69–79. Novosibirsk, SNIIGGiMS (in Russian) (1987).
35. Egorova, L.I., Shabanov, Yu.Ya. & Evtushenko, V.M. (On the stratigraphy of the Perekhod and Sinsk formations in the Lenan Stage stratotype of the Lower Cambrian). *Sibirskiy Nauchno-Issledovatel’skiy Institut Geologii, Geofiziki i Mineral’nogo Syr’ya, Trudy, Seriya Regional’naya Geologiya* **84**, 11–20 (in Russian) (1969).
36. Egorova, L.I., Shabanov, Yu.Ya., Rozanov, A.Yu., Savitskiy, V.E., Chenysheva, N.E. & Shishkin, B.B. (Elanka and Kuonamka facies stratotypes of the lower boundary of the Middle Cambrian in Siberia). *Sibirskiy Nauchno-Issledovatel’skiy Institut Geologii, Geofiziki i Mineral’nogo Syr’ya, Trudy* **211**, 1–167 (in Russian) (1976).
37. Fedorov, A.B. (Biostratigraphy and facies of the oldest Lower Cambrian horizon in the middle reaches of the Aldan River). In: V.A. Astashkiv, ed. (*Stratigraphy and Facies of Sedimentary Basins of Siberia*), pp. 18–25. Novosibirsk, SNIIGGiMS (in Russian) (1982).
38. Fedorov, A.B. (Precambrian and Cambrian boundary strata in the south of the Siberian Platform (analysis of facies conditions)). In, V.I. Krasnov, ed. (*Boundaries of Principal Phanerozoic Subdivision in Siberia*), pp. 29–39. Novosibirsk, SNIIGGiMS (in Russian) (1982).
39. Fedorov, A.B. (New representatives of skeletal organics in the Precambrian-Cambrian stratotype sections of the Siberian Platform (Aldan, Kotuy rivers)). In S.P. Bulynnikova & I.G. Klimova, eds. (*New Species of Ancient Invertebrates and Plants from Oil-Gas-Bearing Provinces of Siberia*), pp. 5–9, 89–90. Novosibirsk, SNIIGGiMS (in Russian) (1984).
40. Fedorov, A.B. (New tubicolous problematics from the Tommotian Stage stratotype). *Paleontologicheskiy Zhurnal* **1986 (2),** 110–111 (in Russian) (1986).
41. Fedorov, A.B. & Shishkin, B.B. (Lower boundary of the Cambrian in the north of the Siberian Platform). In: V.I. Krasnov, ed. (*Problems of the Stage Subdivision of the Phanerozoic Systems in Siberia),* pp. 5–14. Novosibirsk, SNIIGGiMS (in Russian) (1984).
42. Golubev, S.N. (Ontogenic changes and evolutionary trends in the Early Cambrian spiral gastropods Pelagiellacea). *Paleontologicheskiy Zhurnal* **1976 (2),** 34–40 (in Russian) (1976).
43. Goryaeva, I.E. (Trilobites from the Keteme Formation of the Toyonian Stage, Lower Cambrian of the Siberian Platform]. In I.V. Budnikov & B.G. Kraevsky, eds. (*Regional Geology, Stratigraphy and Palaeontology of the Precambrian and Lower Palaeozoic of Siberia*), pp. 53–74. Novosibirsk, SNIIGGiMS (in Russian) (2010).
44. Goryanskiy, V.Yu. (New Early Cambrian obollelids of eastern Siberia). In G.A. Stukalina, ed. (*New Species of Ancient Plants and Invertebrates of the USSR*, Issue 4), pp. 99–102. Moscow, Nauka (in Russian) (1977).
45. Goryanskiy, V.Yu., Egorova, L.I. & Savitskiy, V.E. (On the Lower Cambrian fauna of the northern slope of the Anabar Shield). In N.A. Shvedov, ed. (*Scientific Reports. Series Palaeontology and Biostratigraphy*, Issue 4), pp. 5–32. Leningrad, NIIGA (in Russian) (1964).
46. Gubanov, A.P., Kouchinsky, A.V., Peel, J.S. & Bengtson, S. Middle Cambrian molluscs of ‘Australian type’ from northern Siberia. *Alcheringa* **28**, 1–20 (2004).
47. Gubanov, A.P. & Peel, J.S. *Oelandiella*, the earliest Cambrian helcionellod mollusc from Siberia. *Palaeontology* **42**, 211–222 (1999).
48. Gubanov, A.P. & Peel, J.S. The earliest Cambrian helcionellod mollusc *Anabarella* Vostokova. *Palaeontology* **46**, 1073–1087 (2003).
49. Ivantsov, A.Yu. (First finds of phyllocarids in the Lower Cambrian of Yakutia). *Paleontologicheskiy Zhurnal* **1990 (2),** 130–132 (in Russian) (1990).
50. Ivantsov, A.Yu. (On the finds of typical Ediacaran fossils in the Vendian Yudoma Group of eastern Siberia). *Doklady Akademii nauk* **472**, 1–4 (in Russian) (2017).
51. Ivantsov, A.Yu., Zhuravlev, A.Yu., Krassilov, V.A., Leguta, A.V., Mel’nikova, L.M., Urbanek, A., Ushatinskaya, G.T. & Malakhovskaya, Ya.E.. [Unique Sinsk Localities of Early Cambrian Organisms (Siberian Platform)]. *Paleontologicheskiy Institut Rossiyskoy Academii Nauk, Trudy* **284**, 1–143 (in Russian) (2005).
52. Karlova, G.A. & Vodanyuk, S.A. [New data on transitional strata to the Cambrian in the Khorbusuonka River basin (Olenek Uplift)]. In V.V. Khomentovsky, ed. (*Late Precambrian and Early Palaeozoic Stratigraphy of Siberia: Vendian and Riphean*), pp. 3–13. Novosibirsk, IGiG SO AN SSSR (in Russian) (1985).
53. Khomentovsky, V.V. The Yudomian of Siberia, Vendian and Ediacaran systems of the International Stratigraphic Scale. *Stratigraphy and Geological Correlation* 16, 581–598 (2008).
54. Khomentovskiy, V.V. & Karlova, G.A. (On the lower boundary of the Pestrotsvet Formation in the Aldan River basin). In V.V.Khomentovskiy, ed. (*Late Precambrian and Early Palaeozoic of Siberia. Siberian Platform and the Outer Zone of the Sayan-Altay Fold Belt*), p. 3–22. Novosibirsk, IGiG SO AN SSSR (in Russian) (1986).
55. Khomentovskiy, V.V. & Karlova, G.A. (New data on a correlation of the Vendian-Cambrian strata in eastern and transitional facies regions of Yakutia). In V.V.Khomentovskiy, ed. (*Late Precambrian and Early Palaeozoic of Siberia. Siberian Platform and Its Outskirts*), p. 3–44. Novosibirsk, IGiG SO AN SSSR (in Russian) (1991).
56. Khomentovsky, V.V. & Karlova, G.A. (The Cambrian lower boundary in its grounding in Siberia). *Geologiya i Geofizika* **1992 (4),** 3–26 (in Russian) (1992).
57. Khomentovsky, V.V. & Karlova, G.A. Biostratigraphy of the Vendian–Cambrian beds and the lower Cambrian boundary in Siberia. *Geological Magazine* **130**, 29–45 (1993).
58. Khomentovsky, V.V. & Karlova, G.A. The boundary between Nemakit-Daldynian and Tommotian stages (Vendian–Cambrian systems) of Siberia. *Stratigraphy and Geological Correlation* **10,** 217–238 (2002).
59. Khomentovskii, V.V. & Karlova, G.A. The Tommotian Stage base as the Cambrian lower boundary in Siberia. *Stratigraphy and Geological Correlation* **13**, 21–34 (2005).
60. Khomentovsky, V.V. & Repina, L.N. (*The Lower Cambrian of the Stratotype Section of Siberia*). Moscow, Nauka. 200 p. (in Russian) (1965).
61. Khomentovsky, V.V., Shenfil’, V.Yu., Yakshin, M.S. & Butakov, S.P. (The reference sections of the Upper Precambrian and Lower Cambrian strata on the Siberian Platform). *Institut Geologii i Geofiziki Sibirskogo Otdeleniya Akademii Nauk SSSR,* *Trudy* **141**, 1–356 (in Russian) (1972).
62. Khomentovsky, V.V., Didenko, A.N. & Pyatiletov, V.G. (General conclusions on the Vendian stratigraphy in the western Prianabar’e). In V.V. Khomentovsky, ed. (*New Data on the Late Precambrian Stratigraphy of Siberia*), pp. 3–20. Novosibirsk, IGiG SO AN SSSR (in Russian) (1982).
63. Khomentovsky, V.V., Val’kov, A.K., Karlova, G.A. & Nuzhnov, S.V. (Key section of the Precambrian-Cambrian strata of the Gonam River). In V.V. Khomentovsky, ed. (*Late Precambrian and Early Palaeozoic of Siberia. Vendian Strata*), pp. 29–44. Novosibirsk, IGiG SO AN SSSR (in Russian) (1983).
64. Khomentovsky, V.V., Val’kov, A.K. & Karlova, G.A. (New data on the biostratigraphy of transitional Vendian–Cambrian strata in the middle reaches of the Aldan River). In V.V. Khomentovsky & A.S. Gibsher, eds. (*Late Precambrian and Early Palaeozoic of Siberia. Problems of Regional Stratigraphy*), pp. 3–57. Novosibirsk, IGiG SO AN SSSR (in Russian) (1991).
65. Kochnev, B.B. & Karlova, G.A. New data on biostratigraphy of the Vendian Nemakit-Daldynian Stage in the southern Siberian Platform. *Stratigraphy and Geological Correlation* **18**, 492–504 (2005).
66. Kokoulin, M.L., Zinchenko, V.N., Vasil’eva, N.I., Zazhigin, S.V., Rudavskaya, V.A. & Burova, I.A. (Key section of Vendian and Cambrian strata of the Yakutian Uplift). In Yu.L. Slastenov, ed. (*Regional Geology and Mineral Resources of Yakutia*), pp. 3–15. Yakutsk, Yakutskoe Knizhnoe Izdatel’stvo (in Russian) (1991).
67. Kontorovich, A.E., Varlamov, A.I., Grazhdankin, D.V., Karlova, G.A., Klets, A.G., Kontorovich, V.A., Saraev, S.V., Terleev, A.A., Belyaev, S.Yu., Varaksina, I.V., Efimov, A.S., Kochnev, B.B., Nagovitsin, K.E., Postnikov, A.A. & Filippov, Yu.F.. A section of Vendian in the east of West Siberian Plate (based on data from the Borehole Vostok 3). *Russian Geology and Geophysics* **49,** 932–939 (2008).
68. Korde, K.B. (Problematic fossils from Cambrian strata of the southeastern Siberian Platform). *Doklady Akademii Nauk SSSR* **125**, 625–627 (in Russian) (1959).
69. Korobov, M.N. (New trilobites from the Lower Cambrian of the Kharaulakh Mountains). *Paleontologicheskiy Zhurnal* **1963 (4),** 64–75 (in Russian) (1963).
70. Korobov, M.N. (New trilobites from the Lower Cambrian of Yakutia). *Paleontologicheskiy Zhurnal* **1966 (2),** 57–66 (in Russian) (1966).
71. Korobov, M.N. (New trilobites of the family Conocoryphidae from the Cambrian of the Siberian Platform). *Paleontologicheskiy Zhurnal* **1966 (4),** 92–97 (in Russian) (1966).
72. Korobov, M.N. (Trilobites of the family Conocoryphidae and their significance for the stratigraphy of Cambrian strata). *Geologicheskiy Institut Akademii Nauk SSSR, Trudy* **211**, 1–161 (in Russian) (1973).
73. Korovnikov, I.V. Early and Middle Cambrian phylogeny of Acrothelidae brachiopods. *Russian Geology and Geophysics* **39**, 94–99 (1998).
74. Korovnikov, I.V. Lower and Middle Cambrian boundary and trilobites from northeast Siberian Platform. *Palaeoworld* **13**, 270–275 (2001).
75. Korovnikov, I.V. New data on biostratigraphy of the Lower and Middle Cambrian Series in the northeastern Siberian Platform. *Russian Geology and Geophysics* **43,** 826–836 (2002).
76. Korovnikov, I.V. Lower-Middle Cambrian boundary in open shelf facies of the Siberian Platform. *Palaeoworld* **15**, 424–430 (2006).
77. Korovnikov, I.V. Trilobites of the suborder Eodiscina from the Lower Cambrian of the northeastern Siberian Platform (Khorbosuonka River section). *Paleontological Journal* **41**, 614–620 (2007).
78. Korovnikov, I.V. The lower boundary of the Toyonian stage (Cambrian) of the Siberian Platform. *Russian Geology and Geophysics* **52,** 717–724 (2011).
79. Korovnikov, I.V. & Novozhilova, N.V. New biostratigraphical constraints on the Lower and lower Middle Cambrian of the Kharaulakh Mountains (northeastern Siberian Platform, Chekurovka anticline). *Russian Geology and Geophysics* **53**, 776–786 (2012).
80. Korovnikov, I.V. & Shabanov, Yu.Ya. (Lingulates (brachiopods) from the Middle Cambrian of southeastern Prianabar’e (borehole KCC-2, 519)). *Novosti Paleontologii i Stratigrafii* **2008 (10-11),** 132–137 (in Russian) (2008).
81. Korovnikov, I.V. & Shabanov, Yu.Ya. Trilobites and biostratigraphy of the

Kuonamka Formation, northern Siberian Platform (Olenek River). *Russian Geology and Geophysics* **57**, 562–573 (2016).

1. Korovnikov, I.V., Rowland, S.M., Luchinina, V.A., Shabanov, Yu.Ya. & Fedoseev,

A.V. Biostratigraphy of the Vendian, Lower and Middle Cambrian section of the Enisey River in the Plakhinskiy Island area (north-west of the Siberian Platform). *Russian Geology and Geophysics* **43**, 334–342 (2002).

1. Korshunov, V.I. (*Gonamispongia*—new sponge genus of the family

Chancelloriidae). *Paleontologicheskiy Zhurnal* **1968 (3),** 127–129 (in Russian)

(1968).

1. Korshunov, V.I. (*Lower Cambrian Biostratigraphy and Archaeocyaths of the Northeastern Aldan Anticline*). Yakutsk, Yakutskoe Knizhnoe Izdatel’stvo. 128 pp (in Russian) (1972).
2. Korshunov, V.I. & Zhuravleva, I.T. (New archaeocyath species from the Lower Cambrian of Yakutia). In A.B. Ivanovskiy & B.S. Sokolov, eds. (*New Data on the Lower Palaeozoic Biostratigraphy of the Siberian Platform),* p. 3–11. Novosibirsk, Nauka (in Russian) (1967).
3. Kouchinsky, A. Shell microstructure in early Cambrian molluscs. Acta *Palaeontologica Polonica* **45**, 119–150 (2000).
4. Kouchinsky, A. & Bengtson, S. X-ray tomographic microscopy tightens affinity of the early Cambrian *Oymurania* to the brachiopod stem group. *Acta Palaeontologica Polonica* **62**, 39–43 (2017).
5. Kouchinsky, A., Bengtson, S., Pavlov, V., Runnegar, B., Val’kov, A. & Young, E. Pre-Tommotian age of the lower Pestrotsvet Formation in the Selinde section on the Siberian Platform: Carbon isotope evidence. *Geological Magazine* **142,** 319–325 (2005).
6. Kouchinsky, A., Bengtson, S., Pavlov, V., Runnegar, B., Torssander, P., Young, E. & Ziegler, K. Carbon isotope stratigraphy of the Precambrian–Cambrian Sukharikha River section, northwestern Siberian platform. *Geological Magazine* **144**, 1–10 (2007).
7. Kouchinsky, A., Bengtson, S., Feng, W., Kutygin, R. & Val’kov, A. The Lower Cambrian fossil Anabaritids: affinities, occurrences and systematics. *Journal of Systematic Palaeontology* **7**, 241–298 (2009).
8. Kouchinsky, A., Bengtson, S. & Murdock, D.E.J.A new tannuolinid problematic from the lower Cambrian of the Sukharikha River in northern Siberia*. Acta Palaeontologica Polonica* **55**, 321–331 (2010).
9. Kouchinsky, A., Bengtson, S., Clausen, S., Gubanov, A., Malinky, J.M. & Peel, J.S. A middle Cambrian fauna of skeletal fossils from the Kuonamka Formation, northern Siberia. *Alcheringa* **35**, 123–189 (2011).
10. Kouchinsky, A., Bengtson, S., Runnegar, B., Skovsted, C., Steiner, M. & Vendrasco, M. Chronology of early Cambrian biomineralization. *Geological Magazine* **149**, 221–251 (2012).
11. Kouchinsky, A., Bengtson, S., Clausen, S. & Vendrasco, M.J. An early Cambrian fauna of skeletal fossils from the Emyaksin Formation, northern Siberia. *Acta Palaeontologica Polonica* **60**, 421–512 (2015).
12. Kouchinsky, A., Bengtson, S., Landing, E., Steiner, M., Vendrasco, M. & Ziegler, K. Terreneuvian stratigraphy and faunas from the Anabar Uplift, Siberia. Acta *Palaeontologica Polonica* **62**, 311–440 (2017).
13. Kras’kov, L.N., Lazarenko, N.P., Ogienko, L.V. & Chernysheva N.E. (New Early Palaeozoic trilobites of eastern Siberia and Kazakhstan). In B.P. Markovskiy, ed. (*New Species of Ancient Plants and Invertebrates of the USSR, Part II*), pp. 211–256. Moscow, Gostoptekhizdat (in Russian) (1960).
14. Kruse, P.D., Zhuravlev, A.Yu. & James, N.P. Primordial metazoan-calcimicrobial reefs: Tommotian (Early Cambrian) of the Siberian Platform. *Palaios* **10**, 291–321 (1995).
15. Landing, E. & Kouchinsky, A. Correlation of the Cambrian Evolutionary Radiation: geochronology, evolutionary stasis of earliest Cambrian (Terreneuvian) small shelly fossil (SSF) taxa, and chronostratigraphic significance. *Geological Magazine* (2016).
16. Lazarenko, N.P. (On some Middle Cambrian trilobites of Siberia). *Vestnik LGU, Seriya Biologiya, Geografiya i Geologiya* **1954 (4),** 153–164 (in Russian) (1954).
17. Lazarenko, N.P. (New data on trilobites of the genus *Triangulaspis*). In (*Collection of Papers on Palaeontology and Biostratigraphy*, Issue **3**), pp. 3–17. Leningrad, NIIGA (in Russian) (1957).
18. Lazarenko, N.P. (On the find of *Bathynotus* in the Cambrian deposits of the north of the Siberian Platform). In (*Collection of Papers on Palaeontology and Biostratigraphy*, Issue **8),** pp. 15–19. Leningrad, NIIGA (in Russian) (1958).
19. Lazarenko, N.P. (New Lower Cambrian trilobites from the Soviet Arctic). In N.A. Shvedov, ed. (*Collection of Papers on Palaeontology and Biostratigraphy*, Issue **29**), pp. 29–78. Leningrad, NIIGA (in Russian) (1962).
20. Lazarenko, N.P. (Complexes of Lower Cambrian trilobites from the northern part of central Siberia). *Nauchno-Issledovatel’skiy Institut Geologii Arktiki, Trudy* **137,** 166–287 (in Russian) (1964).
21. Lermontova, E.V. (Class Trilobita). In A.G. Vologdin, ed. (*Atlas of the leading forms of the fossil faunas of the USSR. Volume 1 Cambrian*), pp. 112–193, pl. 35–49. Moscow, Leningrad, Gosudarstvennoe Izdatel’stvo Geologicheskoy Literatury (in Russian) (1940).
22. Lermontova, E.V. (*Lower Cambrian Trilobites and Brachiopods from Eastern Siberia*). Moscow, VSEGEI. 222 p. (in Russian) (1951).
23. Lysova, L.A., Galimova, B.S., Titorenko, T.N. & Fayzulina, Z.K. (Palaeontological characteristics of the Lower Cambrian strata penetrated by the Markovo reference borehole). In (*Geology and Oil-Gas Capacity of Eastern Siberia*), pp. 345–356. Moscow, Nedra (in Russian) (1966).
24. Malakhovskaya, Ya.E. Morphogenesis and evolution of *Kutorgina* Billings, 1861 (Brachiopoda, Kutorginida). *Paleontological Journal* **47**, 11–22.
25. Melnikova, L.M., Siveter, D.J. & Williams, M. Cambrian Bradoriida and Phosphatocopida (Arthropoda) of the former Soviet Union. *Journal of Micropalaeontology* **16**, 179–191.
26. Meshkova, N.P. (Lower Cambrian hyoliths of the Siberian Platform). *Institut Geologii i Geofiziki Sibirskogo Otdeleniya Akademii Nauk SSSR, Trudy* **97**, 1–110 (in Russian).
27. Meshkova, N.P., ed. (Lower and Middle Cambrian biostratigraphy and palaeontology of northern Asia). *Institut Geologii i Geofiziki Sibirskogo Otdeleniya Akademii Nauk SSSR, Trudy* **541**, 1–210 (in Russian) (1983).
28. Meshkova, N.P. & Nikolaeva, I.V., eds. [Precambrian and Cambrian boundary beds of the Siberian Platform (biostratigraphy, palaeontology, depositional conditions)]. *Institut Geologii i Geofiziki Sibirskogo Otdeleniya Akademii Nauk SSSR, Trudy* **475**, 1–201 (in Russian) (1981).
29. Missarzhevsky, V.V. (First finds of *Lapworthella* in the Lower Cambrian of the Siberian Platform). *Paleontologicheskiy Zhurnal* **1966 (1),** 13–18 (in Russian) (1966).
30. Missarzhevsky, V.V. (Conodonts (?) and phosphatic problematica from the Cambrian of Mongolia and Siberia). In L.P. Tatarinov, ed. (*Invertebrates of the Palaeozoic of Mongolia*), p. 10–19. Nauka, Moscow (in Russian) (1977).
31. Missarzhevsky, V.V. (On the Cambrian and Precambrian boundary beds on the western slope of the Olenek Uplift (Olenek River)). *Byulleten’ Moskovskogo Obshchestva Ispytateley Prirody, Otdel Geologicheskiy* **55**, 23–34 (in Russian) (1980).
32. Missarzhevsky, V.V. (The oldest skeletal fossils and stratigraphy of the Precambrian–Cambrian boundary beds). *Geologicheskiy Institut Akademii Nauk SSSR, Trudy* **443**, 1–237 (in Russian) (1989).
33. Missarzhevsky, V.V. & Grigor’eva, N.V. (New representatives of the order Tommotiida). *Paleontologicheskiy Zhurnal* **1981 (4),** 91–97 (in Russian) (1981).
34. Nagovitsin, K.E., Rogov, V.I., Marusin, V.V., Karlova, G.A., Kolesnikov, A.V., Bykova, N.V. & Grazhdankin, D.V. Revised Neoproterozoic and Terreneuvian stratigraphy of the Lena-Anabar Basin and north-western slope of the Olenek Uplift, Siberian Platform. *Precambrian Research* *270,* 226–245 (2015).
35. Naimark, E., Shabanov, Yu. & Korovnikov, I. Cambrian trilobite *Ovatorycocara* Tchernysheva, 1962 from Siberia. *Bulletin of Geosciences* **86**, 405–422 (2011).
36. Netskaya, A.I. & Ivanova, V.A. (The first find of an ostracod in the Lower Cambrian of eastern Siberia). *Doklady AN SSSR* **111**, 1095–1097 (in Russian) (1956).
37. Ogienko, L.V. (Middle Cambrian biostratigraphy and trilobites of the Daldyn-Alakit area of Yakutia). In G.L. Mitrofanov, ed. (Stratigraphy and Biostratigraphy of the South of Eastern Siberia), pp. 14–37. Irkutsk, *Vostochno-Sibirskiy Nauchno-Issledovatel’skiy Institut Geologii, Geofiziki i Mineral’nogo Syr’ya* (in Russian) (1991).
38. Ogienko, L.V. & Garina, S.Y. (*Stratigraphy and Trilobites of the Cambrian of the Siberian Platform).* Moscow, Nauchniy Mir. 380 p. (in Russian).
39. Ogienko, L.V., Byaliy, V.I. & Kolosnitsyna, G.R. (*Biostratigraphy of the Cambrian and Ordovician Deposits of the South of the Siberian Platform*). Moscow, Nedra. 207 p. (in Russian) (2001).
40. Palmer, A.R. & Repina, L.N. Through a glass darkly: Taxonomy, phylogeny, and biostratigraphy of the *Olenellina*. *University of Kansas Paleontological Contributions, New series* **3**, 1–35 (1990).
41. Parkhaev, P.Yu. Two new species of the Cambrian helcionelloid mollusks from the northern part of the Siberian Platform. *Paleontological Journal* **39**, 615–619 (2005).
42. Parkhaev, P.Yu. (Adaptive radiation of Cambrian helcionelloid mollusks (Gastropoda, Archaeobranchia)). In S.V. Rozhnov, ed. (The Evolution of Biosphere and Biodiversity), pp. 282–296. *Moscow, Tovarishchestvo nauchnykh izdaniy KMK* (in Russian) (2006).
43. Parkhaev, P.Yu.. On the genus *Auricullina* Vassiljeva, 1998 and shell pores of the Cambrian helcionelloid mollusks. *Paleontological Journal* **40**, 20–33 (2006).
44. Parkhaev, P.Yu. New data on the morphology of ancient gastropods of the genus *Aldanella* Vostokova, 1962 (Archaeobranchia, Pelagielliformes). *Paleontological Journal* **40**, 244–252 (2006).
45. Parkhaev, P.Yu. 2013. *Carinopelta* nom. nov. and Carinopeltidae nom. nov.—new substitute names for a genus and family of Cambrian gastropods. *Paleontological Journal* **47**, 454 (2013).
46. Parkhaev, P.Yu. *Davidonia* nom. nov.—a new substitute name for a genus of Cambrian gastropods. *Paleontological Journal* **51,** 574 (2017).
47. Parkhaev, P.Yu. Origin and early evolution of the phylum Mollusca. *Paleontological Journal* **51**, 91–112 (2017).
48. Parkhaev, P.Yu. & Karlova, G.A. Taxonomic revision and evolution of Cambrian molluscs of the genus *Aldanella* Vostokova, 1962 (Gastropoda: Archaeobranchia). *Paleontological Journal* **45**, 1145–1205 (2011).
49. Parkhaev, P.Yu., Karlova, G.A. & Rozanov, A.Yu. Stratigraphic distribution of two potential index species for the GSSP of Cambrian Stage 2 – *Aldanella attleborensis* and *Watsonella crosbyi*. In Y. Zhao, M. Zhu, J. Peng, R.R. Gaines & R.L. Parsley, eds. Cryogenian – Ediacaran to Cambrian Stratigraphy and Paleontology of Guizhou, China. *Journal of Guizhou University, Natural Sciences*, 179–180 (2012).
50. Pegel’, T.V. (New trilobites from the Lower Cambrian of Siberia). In V.S. Surkov, ed. (*New Species of Ancient Invertebrates and Plants from the Oil and Gas Provinces of Siberia*), pp. 15–19. Novosibirsk, SNIIGGiMS (in Russian) (1984).
51. Pegel’, T.V. (Trilobites of the Middle Cambrian Tangha-Ust’Mil’ reefal complex of the Siberian Platform). In S.P. Bulynnikova & I.G. Klimova, eds. *(New Species of Ancient Plants and Invertebrates from the Phanerozoic of Siberia*), pp. 75–79. Novosibirsk, SNIIGGiMS (in Russian) (1987).
52. Pegel, T.V. Evolution of trilobite biofacies in Cambrian basins of the Siberian Platform. *Journal of Paleontology* **74**, 1000–1019 (2000).
53. Pegel’, T.V. & Khramova, A.P. (Trilobites of the Cambrian Chukuka lithofacies complex of the Siberian Platform). In V.A. Astashkin, ed. (*Stratigraphy and Palaeontology of the Precambrian and Phanerozoic of Siberia),* pp. 37–46. Novosibirsk, SNIIGGiMS (in Russian) (1985).
54. Pegel’, T.V., Еgorovа, L.I., Shabanov, Yu.Ya., Korovnikov, I.V., Luchinina, V.А., Salikhova, А.К., Sundukov, V.М., Fedorov, А.B., Zhuravlev, А.Yu., Parkhaev, P.Yu., Demidenko, Yu.Е. (Stratigraphy of Oil and Gas Basins of Siberia. Cambrian of Siberian Platform. V. 2 – Paleontology). *Novosibirsk, IPGG SB RAS.* 344 pp (in Russian) (2016).
55. Pel’man, Yu.L. 1977. (Early and Middle Cambrian inarticulate brachiopods of the Siberian Platform). *Institut Geologii i Geofiziki Sibirskogo Otdeleniya Akademii Nauk SSSR, Trudy* **316**, 1–168 (in Russian) (1977).
56. Pel’man, Yu.L., Aksarina, N.A., Koneva, S.P. Popov, L.E., Sobolev, L.P. & Ushatinskaya, G.T. (The oldest brachiopods from the territory of northern Eurasia). *Novosibirsk, OIGGiM SO RAN.* 145 p. (in Russian) (1992).
57. Pokrovskaya, N.V. (Stratigraphy of the Cambrian sediments in the south of the Siberian Platform). In N.S. Shatskiy, ed. (Problems of Geology in Asia, Volume 1), pp. 444–465. *Moscow, Izdatel’stvo AN SSSR* (in Russian) (1954).
58. Pokrovskaya, N.V. (Middle Cambrian agnostids of Yakutia). *Geologicheskiy Institut Akademii Nauk SSSR, Trudy* **16**, 1–96 (in Russian) (1958).
59. Repina, L.N. (*Lower Cambrian Trilobites from the South of Siberia (Superfamily Redlichioidea, Part I*). Moscow, Nauka. 203 p. (in Russian) (1966).
60. Repina, L.N. (*Lower and Middle Cambrian Trilobites from the South of Siberia (Superfamily Redlichioidea, Part II)).* Moscow, Nauka. 109 p. (in Russian) (1969).
61. Repina, L.N. (Evolution of trilobites at the early stages of their historical development). *Institut Geologii i Geofiziki Sibirskogo Otdeleniya Akademii Nauk SSSR, Trudy* **764**, 34–44 (in Russian) (1990).
62. Repina, L.N., ed. (*Cambrian Biostratigraphy and Palaeontology of Northern Asia*). Novosibirsk, Nauka. 222 pp. (in Russian) (1990).
63. Repina, L.N. & Suvorova, N.P., eds. (Biostratigraphy and fauna of the Lower and Middle Cambrian boundary beds in Siberia). *Institut Geologii i Geofiziki Sibirskogo Otdeleniya Akademii Nauk SSSR, Trudy* **548**, 1–136 (in Russian) (1983).
64. Repina, L.N., Lazarenko, N.P., Meshkova, N.P., Korshunov, V.T., Nikiforov, N.T. & Aksarina, N.A. [Biostratigraphy and fauna of the Lower Cambrian of the Kharulakh (Tuora-Sis Ridge)]. *Institut Geologii i Geofiziki Sibirskogo Otdeleniya Akademii Nauk SSSR, Trudy* **235**, 1–299 (in Russian) (1974).
65. Riding, R. & Zhuravlev, A.Yu. Structure and diversity of oldest sponge-microbe reefs: Lower Cambrian, Aldan River, Siberia. *Geology* **23**, 649–652 (1995).
66. Rowland, S.M., Luchinina, V.A., Korovnikov, I.V., Sipin, D.P., Tarletskov, A.I. & Fedoseev, A.V. Biostratigraphy of the Vendian-Cambrian Sukharikha River section, northwestern Siberian Platform. *Canadian Journal of Earth Sciences* **35**, 339–352 (1998).
67. Rogov, V.I., Karlova, G.A., Marusin, V.V., Kochnev, B.B., Nagovitsin, K.E. & Grazhdankin, D.V. Duration of the first biozone in the Siberian hypostratotype of the Vendian. *Russian Geology and Geophysics* **56**, 573–583 (2015).
68. Rozanov, A.Yu. (Regularities in the morphological evolution of archaeocyaths and problems of Lower Cambrian stage division). *Geologicheskiy Institut Akademii Nauk SSSR, Trudy* **241**, 1–164 (in Russian) (1973).
69. Rozanov, A.Yu. & Missarzhevsky, V.V. (Biostratigraphy and fauna of the Cambrian lower horizons). *Geologicheskiy Institut Akademii Nauk SSSR, Trudy* **148**, 1–127 (in Russian) (1966).
70. Rozanov, A.Yu. & Sokolov, B.S., eds. (*Lower Cambrian Stage Subdivision. Stratigraphy*). Moscow, Nauka. 184 pp (in Russian) (1984).
71. Rozanov, A.Yu. & Zhuravlev, A.Yu. The Lower Cambrian fossil record of the Soviet Union. In J.H. Lipps, & P.W. Signor, eds. *Origin and Early Evolution of the Metazoa*, pp. 205–282. New York, Plenum Press (1992).
72. Rozanov, A.Yu., Missarzhevsky, V.V., Volkova, N.A., Voronova, L.G., Krylov, I.N., Keller, B.M., Korolyuk, I.K., Lendzion, K., Michniak, R., Pykhova, N.G. & Sidorov, A.D. (The Tommotian Stage and the Cambrian lower boundary problem). *Geologicheskiy Institut Akademii Nauk SSSR, Trudy* **206**, 1–380 (in Russian) (1969).
73. Rozanov, A.Yu., Parkhaev, P.Yu., Demidenko, Yu.E., Karlova, G.A., Korovnikov, I.V., Shabanov, Yu.Ya., Ivantsov, A.Yu., Luchinina, V.A., Malakhovskaya, Ya.E., Melnikova, L.M., Naimark, E.B., Ponomarenko, A.G., Skorlotova, N.A., Sundukov, V.M., Tokarev, D.A., Ushatinskaya, G.T. & Kipriyanova, L.K. (*Fossils from the Lower Cambrian Stage Stratotypes*). Moscow, PIN RAN. 228 p. (in Russian) (2010).
74. Rozanov, A.Yu., Repina, L.N., Apollonov, M.K., Shabanov, Yu.Ya., Zhuravlev, A.Yu., Pegel’, T.V., Fedorov, A.B., Astashkin, V.A., Zhuravleva, I.T., Egorova, L.I., Chugaeva, M.N., Dubinina, S.V., Ermak, V.V., Esakova, N.V., Sundukov, V.V., Sukhov, S.S. & Zhemchuzhnikov, V.G. (*The Cambrian of Siberia*). Novosibirsk, Nauka. 135 pp (in Russian) (1992).
75. Rozhnov, S.V. Carpozoan echinoderms from the Middle Cambrian (Mayaktakh Formation) of Siberia (lower reaches of the Lena River). *Paleontological Journal* **40,** 266–275 (2006).
76. Rozhnov, S.V., Fedorov, A.B. & Sayutina, T.A. Lower Cambrian echinoderms from Russia. *Paleontological Journal* **1**, 53–66 (1992).
77. Rozova, A.B. (*Biostratigraphy and Descriptions of Trilobites from the Middle and Upper Cambrian of the North-West of the Siberian Platform*). Moscow, Nauka. 148 p. (in Russian) (1964).
78. Rudavskaya, V.A. & Vasil’eva, N.I. (Acritarchs and skeletal problematics on the Vendian, Tommotian and Atdabanian stage boundaries). In M.L. Kokoulin & V.A. Rudavskaya, eds. (*Late Precambrian and Early Palaeozoic Stratigraphy of the Siberian Platform*), pp. 51–57. Leningrad, VNIGRI (in Russian) (1985).
79. Salikhova, A.K. (New Middle Cambrian trilobites of the Olenek Uplift). In S.P. Bulynnikova & I.G. Klimova, eds. (*New Species of Ancient Plants and Invertebrates from the Phanerozoic of Siberia*), pp. 79–85. Novosibirsk, SNIIGGiMS (in Russian) (1987).
80. Savitskiy, V.E., ed. (Geology of reef systems in the Cambrian of Western Yakutia). *Sibirskiy Nauchno-Issledovatel’skiy Institut Geologii, Geofiziki i Mineral’nogo Syr’ya, Trudy* **270**, 1–151 (in Russian) (1979).
81. Savitskiy, V.E., Shabanov, Yu.Ya. & Shishkin, B.B. (Stratigraphy of Lower and early Middle Cambrian deposits in the Igarka area). *Sibirskiy Nauchno-Issledovatel’skiy Institut Geologii, Geofiziki i Mineral’nogo Syr’ya, Trudy* **32 (2),** 42–62 (in Russian) (1964).
82. Savitskiy, V.E., Shishkin, B.B. & Shabanov, Yu.Ya. (On the stratigraphic subdivision of the Precambrian and Cambrian deposits of the Igarka area). *Sibirskiy Nauchno-Issledovatel’skiy Institut Geologii, Geofiziki i Mineral’nogo Syr’ya, Trudy,* *Seriya Regional’naya Geologiya* **57**, 133–149 (in Russian) (1967).
83. Savitskiy, V.E., Evtushenko, V.M., Egorova, L.L., Kontorovitch, A.E. & Shabanov, Yu.Ya. (Cambrian of the Siberian Platform (Yudoma-Olenek section type, Kuonamka complex deposits)). *Sibirskiy Nauchno-Issledovatel’skiy Institut Geologii, Geofiziki i Mineral’nogo Syr’ya, Trudy* **130,** 1–198 (in Russian) (1972).
84. Sayutina, T.A. (Early Cambrian family Khasaktiidae fam. nov.—possible stromatoporates). *Paleontologicheskiy Zhurnal* **1980 (4),** 13–28 (in Russian) (1980).
85. Shabanov, Yu.Ya., Astashkin, V.A., Pegel, T.V., Egorova, L.I., Zhuravleva, I.T., Pel’man, Yu.L., Sundukov, V.M., Stepanova, M.V., Sukhov, S.S., Fedorov, A.B., Shishkin, B.B., Vaganova, N.V., Ermak, V.I., Ryabukha, K.V., Yadrenkina, A.G., Abaimova, G.P., Lopushinskaya, T.V., Sychev, O.V. & Moskalenko, T.A. (*Lower Palaeozoic of the Anabar Anteclise Southwestern Slope (according to borehole data)).* Novosibirsk, Nauka, 208 pp. (in Russian) (1987).
86. Shabanov, Yu.Ya., Korovnikov, I.V., Pereladov, V.S., Fefelov, A.F., Lazarenko, N.P., Gogin, I.Ya., Pegel, T.V., Sukhov, S.S., Abaimova, G.P., Egorova, L.I., Fedorov, A.B., Raevskaya, E.G. & Ushatinskaya, G.T. *The Cambrian System of the Siberian Platform. Part 2: North-East of the Siberian Platform*. Moscow; Novosibirsk, PIN RAS. 140 pp. (2008).
87. Shishkin, B.B. (Shelly fauna in the Nemakit-Daldyn Formation (northwest of the Anabar uplift)). *Geologiya i Geofizika* **1974 (4),** 111–114 (in Russian) (1974).
88. Shishkin, B.B., Fedorov, A.B. & Sundukov, V.M. (Kotuy archaeocyathan horizon in the southwestern Prioanabar’e). In V.V. Khomentovsky, ed. (*New Data on the Late Precambrian Stratigraphy of Siberia),* pp. 20-30. Novosibirsk, IGiG SO AN SSSR (in Russian) (1982).
89. Skovsted, C.B., Ushatinskaya, G.T., Holmer, L.E., Popov, L.E. & Kouchinsky, A. Taxonomy, morphology, shell structure and ontogeny of *Pelmanotreta* nom. nov. from the lower Cambrian of Siberia. *GFF* **137**, 1–8 (2015).
90. Sokolov, B.S. Vendian and Early Cambrian Sabelliditidae (Pogonophora) of the USSR. In *Proceedings of the 23d International Geological Congress,* pp. 79–86. Prague, IPU (1972).
91. Sokolov, B.S. & Zhuravleva, I.T., eds. (Stage subdivision of the Early Cambrian. Atlas of fossils). *Institut Geologii i Geofiziki Sibirskogo Otdeleniya Akademii Nauk SSSR, Trudy* **558**, 1–216 (in Russian) (1983).
92. Sokolov, B.S. & Zhuravleva, I.T., eds. (Problematics of the Late Precambrian and Palaeozoic). *Institut Geologii i Geofiziki Sibirskogo Otdeleniya Akademii Nauk SSSR, Trudy* **632**, 1–178 (in Russian) (1985).
93. Solov’ev, I.A. (Some new trilobites from the Amgan Stage of the Olenek area of Yakutia), In N.A. Shvedov, ed. (*Scientific Reports. Series Palaeontology and Biostratigraphy*, Issue **4**), pp. 33–55. Leningrad, NIIGA (in Russian) (1964).
94. Solov’ev, I.A (On the Middle Cambrian genus *Prohedinia* (trilobites)). In (Scientific Reports of the Scientific-Research Institute of Geology of Arctic. Palaeontology and Biostratigraphy, Issue 14), pp. 11–27. Leningrad, NIIGA (in Russian) (1966).
95. Solov’ev, I.A. (New species of *Paradoxides* (trilobites) from oil shales of the Amgan Stage of northern Yakutia). In (*Scientific Reports of the Scientific-Research Institute of Geology of Arctic. Palaeontology and Biostratigraphy*, Issue **25**), pp. 9–20. Leningrad, NIIGA (in Russian) (1969).
96. Solov’ev, I.A. (On the find of a representative of the genus *Burlingia* in the Amgan Stage of the north of Siberia), In A.A. Gerke, ed. (*Scientific Reports of the Scientific-Research Institute of Geology of Arctic. Palaeontology and Biostratigraphy,* Issue **26**), pp. 9–12. Leningrad, NIIGA (in Russian) (1969).
97. Solov’ev, I.A. (New trilobites from the Lower Cambrian of the Siberian Platform). *Paleontologicheskiy Zhurnal* **1988 (3),** 56–63 (in Russian) (1988).
98. Sundukov, V.M. (New archaeocyaths from the Lower Cambrian of the Lena and Kotuy). *Paleontologicheskiy Zhurnal* **1983 (4),** 13–17 (in Russian) (1983).
99. Sundukov, V.M. (New species of archaeocyaths from the Lower Cambrian of the south-east of the Siberian Platform). In S.P. Bulynnikova & I.G. Klimova, eds. (*New Species of Ancient Invertebrates and Plants from Oil-Gas-Bearing Provinces of Siberia*), pp. 10–15. Novosibirsk, SNIIGGiMS (in Russian) (1984).
100. Sundukov, V.M. (New species of archaeocyaths from the Lower Cambrian of the West-Yakutian barrier reef complex). In S.P. Bulynnikova & I.G. Klimova, eds. (*New Species of Ancient Plants and Invertebrates from the Phanerozoic of Siberia*), pp. 46–51. Novosibirsk, SNIIGGiMS (in Russian) (1987).
101. Sundukov,V.M & Zhuravlev, A.Yu. (First finds of cribricyaths in the Lower Cambrian of the Siberian Platform). *Paleontologicheskiy Zhurnal* **1989 (3**), 101–102 (in Russian) (1989).
102. Suvorova, N.P.(Cambrian trilobites of the east of the Siberian Platform. Issue 1: Protolenids). *Paleontologicheskiy Institut Akademii Nauk SSSR 6, Trudy,* 1–182 (in Russian) (1956).
103. Suvorova, N.P. (New trilobites from the Lower Cambrian of Yakutia). *Doklady AN SSSR* **122**, 917–920 (in Russian) (1958).
104. Suvorova, N.P. (New trilobites from the superfamilies Corynexochoidea and Redlichoidea of the Lower Cambrian Lenan Stage of Yakutia). *Paleontologicheskiy Zhurnal* **1959 (3),** 65–77 (in Russian) (1959).
105. Suvorova, N.P. (Cambrian trilobites of the east of the Siberian Platform. Issue 2: Olenellids-granulariids). *Paleontologicheskiy Institut Akademii Nauk SSSR* **84,** Trudy, 1–238 (in Russian) (1960).
106. Suvorova, N.P. (Systematic position of some Siberian trilobites). *Paleontologicheskiy Zhurnal* **1981 (2),** 99–109 (in Russian) (1981).
107. Suvorova, N.P. (Corynexochoid trilobites and their historical development). *Paleontologicheskiy Institut Akademii Nauk SSSR* **103**, Trudy, 1–319 (in Russian) (19864).
108. Sysoev, V.A. (Hyoliths of the genus *Circotheca* from the Lower Cambrian of the Taymyr District). *Paleontologicheskiy Zhurnal* **1959 (1),** 84–92 (in Russian) (1959).
109. Sysoev, V.A. (Hyoliths of the genera *Circotheca, Orthotheca* from the Lower Cambrian of the Siberian Platform). *Paleontologicheskiy Zhurnal* **1959 (2),** 68–78 (in Russian) (1959).
110. Sysoev, V.A. (Cambrian Hyoliths from the Northern Slope of the Aldan Shield). *Yakutsk, YaFAN SSSR*. 66 p. (in Russian) (1962).
111. Sysoev, V.A. (Hyoliths of the genus *Torellella* from the Lower Cambrian of the Anabar Anteclise). *Paleontologicheskiy Zhurnal* **1963 (3),** 49–55 (in Russian) (1963).
112. Sysoev, V.A. (To the systematic of the order Camerothecida). In V.F. Vozin, ed. (*Palaeontology and Stratigraphy of Palaeozoic and Triassic Strata of Yakutia*), pp. 21–27. Moscow, Nauka (in Russian) (1965).
113. Sysoev, V.A. (*Brevilabiatus*—a new hyolith genus). In V.F. Vozin, ed. (*Palaeontology and Stratigraphy of Palaeozoic and Triassic Strata of Yakutia*), pp. 28–30. Moscow, Nauka (in Russian) (1965).
114. Sysoev, V.A. (Stratigraphy and Hyoliths of the oldest Lower Cambrian strata of the Siberian Platform). *Yakutsk, Yakutskoe knizhnoe izdatel’stvo*. 67 p. (in Russian) (1968).
115. Sysoev, V.V. (On a new family of Lower Cambrian hyoliths). In A.K. Bobrov, ed. (Stratigraphy and Palaeontology of the Proterozoic and Cambrian on the East of the Siberian Platform), pp. 109–115. *Yakutsk, Yakutskoe knizhnoe izdatel’stvo* (in Russian) (1970).
116. Sysoev, V.A. (*Lower Cambrian Biostratigraphy and Orthothecimorph Hyoliths of the Siberian Platform*). Moscow, Nauka. 152 p. (in Russian) (1972).
117. Tkachenko, V.I., Ushatinskaya, G.T., Zhuravlev A.Yu. & Repina, L.N. 1987. (Cambrian strata of the Kolyma Uplift). *Izvestiya Academii Nauk SSSR, Seriya Geologicheskaya* **1987 (8),** 55–62 (in Russian) (1987).
118. Ushatinskaya, G.T. (Early and Middle Cambrian lingulids of the Siberian Platform). *Paleontologicheskiy Zhurnal* **1993 (2),** 133–136 (in Russian).
119. Ushatinskaya, G.T. (New Middle-Upper Cambrian acrotretids (brachiopods) from the north of the Siberian Platform and some problems of their systematic). *Paleontologicheskiy Zhurnal* **1994 (4),** 38–54 (in Russian).
120. Ushatinskaya, G.T. (The oldest lingulates). *Paleontologicheskiy Institut Rossiyskoy Academii Nauk, Trudy* **262**, 1–91 (in Russian) (1995).
121. Ushatinskaya, G.T. The oldest lingulids of the Siberian Platform: Microornamentation and shell structure. *Paleontological Journal* **46**, 1298–1308 (2012).
122. Ushatinskaya, G.T. & Korovnikov, I.V. Revision of the Early—Middle Cambrian Lingulida (Brachiopoda) from the Siberian Platform. *Paleontological Journal* **48**, 28–41 (2014).
123. Ushatinskaya, G.T. & Korovnikov, I.V. Revision of the superfamily Acrotheloidea (Brachiopoda, class Linguliformea, order Lingulida) from the Lower and Middle Cambrian of the Siberian Platform. *Paleontological Journal* **50**, 450–462 (2016).
124. Ushatinskaya, G.T. & Malakhovskaya, Ya.E. Origin and development of the Cambrian brachiopod biochores. *Stratigraphy and Geological Correlation* **9**, 540–556 (2001).
125. Ushatinskaya, G.T. & Malakhovskaya, Ya.E. (The first brachiopods with a carbonate skeleton: Appearance, migration, shell wall structure). In S.V. Rozhnov, ed. (*The Evolution of Biosphere and Biodiversity*), pp. 177–192. Moscow, Tovarishchestvo nauchnykh izdaniy KMK (in Russian) (2006).
126. Val’kov, A.K. (To the fauna of the Kessyuse Formation of the Lower Cambrian of the Olenek Uplift). In K.B. Mokshantsev, ed. (*Tectonics, Stratigraphy and Lithology of Sedimentary Formations in Yakutia)* , pp. 115–119. Yakutsk, Yakutskoe knizhnoe izdatel'stvo (in Russian) (1968).
127. Val’kov, A.K. (Hyoliths of the Middle Cambrian Amgan Stage from the north-east of the Siberian Platform). In A.K. Bobrov, ed. (*Stratigraphy and Palaeontology of the Proterozoic and Cambrian on the East of the Siberian Platform*), pp. 71–90. Yakutsk, Yakutskoe knizhnoe izdatel’stvo (in Russian) (1970).
128. Val’kov, A.K. (*Biostratigraphy and Hyoliths of the Cambrian of Northeastern Siberian Platform).* Moscow, Nauka. 139 p. (in Russian).
129. Val’kov, A.K. (*Biostratigraphy of the Lower Cambrian in the East of the Siberian Platform (Utchur-Maya Region)).* Moskva, Nauka. 92 p. (in Russian).
130. Val’kov, A.K. (Distribution of the oldest skeletal organisms and a correlation of the lower Cambrian boundary in the south-eastern part of the Siberian Platform). In V.V. Khomentovsky, ed. (*Late Precambrian and Early Palaeozoic of Siberia. Vendian Strata*), pp. 37–48, 88–90. Novosibirsk, IGiG SO AN SSSR (in Russian) (1983).
131. Val’kov, A.K. (*Biostratigraphy of the Lower Cambrian of Eastern Siberian Platform (Yudoma-Olenek Region)).* Nauka, Moscow. 136 p. (in Russian) (1987).
132. Val’kov, A.K. & Bokova, A.R. (Composition and geological distribution of the Sunnaginian fossils on the northern slope of the Aldan Anteclise). *Geologiya i Geofizika* **1989 (7),** 133 (in Russian) (1989).
133. Val’kov, A.K. & Karlova, G.A. (Fauna from transitional Vendian-Cambrian strata in the lower reaches of the Gonam River). In V.V. Khomentovsky, ed. (*Late Precambrian and Early Palaeozoic Stratigraphy: Central Siberia*), pp. 12–41. Novosibirsk, IGiG SO AN SSSR (in Russian) (1984).
134. Val’kov, A.K. & Sysoev, V.V. (Cambrian angustiochreids of Siberia). In A.K. Bobrov, ed. (*Stratigraphy and Palaeontology of the Proterozoic and Cambrian on the East of the Siberian Platform*), pp. 94–100. Yakutsk, Yakutskoe knizhnoe izdatel’stvo (in Russian) (1970).
135. Varlamov, A.I. & Sundukov, V.M. (Archaeocyathan-algal bioherms of the Olekma Formation of the Lower Cambrian on the Amga River). In L.N. Repina & I.T. Zhuravleva, eds. (*News in the Lower Palaeozoic Stratigraphy and Palaeontology of Central Siberia),* pp. 27–35. Novosibirsk, IGiG SO AN SSSR (in Russian) (1978).
136. Varlamov, A.I., Rozanov, A.Yu., Khomentovskiy, V.V., Shabanov, Yu.Ya., Abaimova, G.P., Demidenko, Yu.E., Karlova, G.A., Korovnikov, I.V., Luchinina, V.A., Malakhovskaya, Ya.E., Parkhaev, P.Yu., Pegel, T.V., Skorlotova, N.A., Sundukov, V.M., Sukhov, S.S., Fedorov, A.B. & Kipriyanova, L.K. *The Cambrian System of the Siberian Platform. Part 1: The Aldan-Lena Region.* Moscow; Novosibirsk, PIN RAS. 300 pp. (2008).
137. Vasil’eva, N.I. [Biostratigraphy of the Cambrian lower horizons in north-eastern part of the Siberian Platform (lower Olenek River; Lena River; Udzha River)]. In M.L. Kokoulin & V.A. Rudavskaya, eds. (*Late Precambrian and Early Palaeozoic Stratigraphy of the Siberian Platform*), pp. 5–15. Leningrad, VNIGRI (in Russian) (1985).
138. Vasil’eva, N.I. (New anabaritid genus from the Lower Cambrian of the Siberian Platform). *Paleontological Zhurnal* **1986 (2),** 103–104 (in Russian) (1986).
139. Vasil’eva, N.I. (New Early Cambrian gastropods of the Siberian Platform). In A.I. Nikolaev, ed. (*Microfauna of the USSR. Problems of the Systematics and Biostratigraphy*), pp. 4–21. Leningrad, VNIGRI (in Russian) (1990).
140. Vasil’eva, N.I. (Early Cambrian small shelly fauna from boreholes of western Yakutia). *Paleontological Zhurnal* **1994 (4),** 3–9 (in Russian) (1994).
141. Vasil’eva, N.I. (*Small Shelly Fauna and Biostratigraphy of the Lower Cambrian of the Siberian Platform*). St. Petersburg, VNIGRI. 139 pp (in Russian) (1998).
142. Vasil’eva, N.I. & Rudavskaya, V.A. [Regularities in the distribution of fauna and phytoplankton communities in the Vendian and Cambrian boundary beds on the Siberian Platform). In M.S. Messezhnikov & S.A. Chirva, eds. (*Methodical Aspects of Stratigraphical Studies in Oil-Gas-Bearing Basins*), pp. 69–79. Leningrad, VNIGRI (in Russian) (1991).
143. Vasil’eva, N.I. & Sayutina, T.A. (New genus and species names for Early Cambrian chancelloriid sclerites). *Paleontological Zhurnal* **1993 (1),** 113–114 (in Russian) (1993).
144. Vodanyuk, S.A. & Karlova, G.A. (On the Kessyuse Formation of the Olenek Uplift). In V.V. Khomentovsky & V.Yu. Shenfil’, eds. (*Late Precambrian and Early Palaeozoic of Siberia: Riphean and Vendian*), pp. 3–20. Novosibirsk, IGiG SO AN SSSR (in Russian) (1988).
145. Voronin, Yu.I. (Ajacicyathids of the USSR). *Paleontologicheskiy Institut Akademii Nauk SSSR, Trudy* **176,** 1–148 (in Russian) (1979).
146. Voronova, L.G. & Missarzhevsky, V.V. 1969. (Finds of algae and worm tubes in the Cambrian and Precambrian boundary strata on the north of the Siberian Platform). *Doklady Akademii Nauk SSSR* **184**, 204–210 (in Russian) (1969).
147. Vostokova,V.A. 1962. (The Cambrian gastropods from Siberia and Taimyr). In N.A. Shvedov, ed. (*Collection of Papers on Palaeontology and Biostratigraphy,* Issue **28**), pp. 51–74. Leningrad, NIIGA (in Russian) (1962).
148. Whittington, H.B., Chatterton, B.D.E., Speyer, S.E., Fortey, R.A., Owens, R.M., Chang, W.T., Dean, W.T., Jell, P.A., Laurie, J.R., Palmer, A.R., Repina, L.N., Rushton, A.W.A., Shergold, J.H., Clarkson, E.N.K., Wilmot, N.V. & Kelly, S.R.A. *Treatise on Invertebrate Paleontology, Part O (Revised),* Trilobita, Introduction, Order Agnostina, Order Redlichiida. Boulder, Colorado, Geological Society of America; Lawrence, Kansas, University of Kansas. 530 pp (1997).
149. Williams, M., Siveter, D.J., Popov, L.E. & Vannier, J.M.C. Biogeography and affinities of bradoriid arthropods: Cosmopolitan microbenthos of the Cambrian seas. *Palaeogeography, Palaeoclimatology, Palaeoecology* **248,** 202–232 (2007).
150. Zhu, M., Zhuravlev, A.Yu., Wood, R.A., Zhao, F. & Sukhov, S.S. A deep root for the Cambrian Explosion: Implications of new bio- and chemostratigraphy from the Siberian Platform. *Geology* (2017).
151. Zhuravlev, A.Yu.. Biota diversity and structure during the Neoproterozoic-Ordovician transition. In A.Yu. Zhuravlev & R. Riding, eds. *The Ecology of the Cambrian Radiation*, pp. 173–199. New York, Columbia University Press. (2001).
152. Zhuravlev, A.Yu. (Features of the diversification of organisms in the Cambrian). In A.G. Ponomarenko, A.Yu. Rozanov & M.A. Fedonkin, eds. (*Ecosystem Restructures and the Evolution of the Biosphere*, Issue 4), p. 174–183. Moscow, Paleontologicheskiy Institut Rossiyskoy Akademii Nauk (in Russian) (2001).
153. Zhuravlev, A.Yu., Debrenne, F. & Lafuste, J. 1993. Early Cambrian microstructural diversification of Cnidaria. *Courier Forschungsinstitut Senckenberg* **164,** 365–372 (1993).
154. Zhuravlev, A.Yu., Naimark, E.B. & Wood, R.A. 2015. Controls on the diversity and structure of earliest metazoan communities: early Cambrian reefs from Siberia. *Earth-Science Reviews* **147**, 18–29 (2015).
155. Zhuravlev, A.Yu. & Repina, L.N., eds. Guidebook for excursion on the Aldan and Lena Rivers. Siberian Platform. *3rd Internat. Symp. Cambrian System*. IGIG, Novosibirsk, 115 pp. (1990).
156. Zhuravlev, A.Yu., & Wood, R. Lower Cambrian reefal cryptic communities. *Palaeontology* **18**, 443–470 (1995).
157. Zhuravlev, A.Yu. & Wood, R.A. Eve of biomineralization: Controls on skeletal mineralogy. *Geology* **36,** 923–926 (2008).
158. Zhuravleva, I.T. (*Archaeocyaths of the Siberian Platform*). Moscow, Akademiya Nauk SSSR. 344 p. (in Russian) (1960).
159. Zhuravleva, I.T., ed. (*Lower Cambrian Biostratigraphy and Palaeontology of Siberia and the Far East*). Moscow, Nauka. 288 pp (in Russian) (1969).
160. Zhuravleva, I.T., ed. (*Problems of Lower Cambrian Biostratigraphy and Paleontology of Siberia).* Moscow, Nauka. 228 pp (in Russian) (1972).
161. Zhuravleva, I.T., ed. (*Problems of Lower Cambrian Paleontology and Biostratigraphy of Siberia and the Far East*). Novosibirsk, Nauka. 266 pp (in Russian) (1973).
162. Zhuravleva, I.T., ed. (Lower and Middle Cambrian Stratigraphy and Palaeontology of the U.S.S.R.). *Institut Geologii i Geofiziki Sibirskogo Otdeleniya Akademii Nauk SSSR, Trudy* **296**, 258 р. (in Russian) (1976).
163. Zhuravleva, I.T., ed. (Cambrian Biostratigraphy and Palaeontology of Northern Asia). *Institut Geologii i Geofiziki Sibirskogo Otdeleniya Akademii Nauk SSSR, Trudy* **669**, 1–230 (in Russian) (1986).
164. Zhuravleva, I.T. & Meshkova, N.P., eds. (Lower Cambrian Biostratigraphy and Palaeontology of Siberia). *Institut Geologii i Geofiziki Sibirskogo Otdeleniya Akademii Nauk SSSR, Trudy* **406**, 1–160 (in Russian) (1979).
165. Zhuravleva, I.T. & Repina, L.N., eds. (Cambrian of Siberia and Central Asia). *Institut Geologii i Geofiziki Sibirskogo Otdeleniya Akademii Nauk SSSR*, *Trudy* **720,** 1–210 (in Russian) (1988).
166. Zhuravleva, I.T. & Rozanov A.Yu., eds. (*Lower Cambrian Biostratigraphy and Paleontology of Europe and Northern Asia*). Moscow, Nauka. 312 рp (in Russian) (1974).
167. Zinchenko, V.N., Vasil’eva, N.I. & Rudavskaya, V.A. (Basal Cambrian strata on the north-eastern margin of the Siberian Platform). In S.A. Chirva & V.N. Zinchenko, eds. (*Phanerozoic Stratigraphy of Oil-Gas-Bearing Regions of Russia*), pp. 4–18. St.Petersburg, VNIGRI (in Russian) (1993).
